# Studying the “Rigid–Flexible” Properties of Polymeric Micelle Core-Forming Segments with a Hydrophobic Phthalocyanine Probe Using NMR and UV Spectroscopy

**DOI:** 10.1021/acs.langmuir.1c00328

**Published:** 2021-04-02

**Authors:** Łukasz Lamch, Roman Gancarz, Marta Tsirigotis-Maniecka, Izabela M. Moszyńska, Justyna Ciejka, Kazimiera A. Wilk

**Affiliations:** Department of Engineering and Technology of Chemical Processes, Faculty of Chemistry, Wrocław University of Science and Technology, Wybrzeże Wyspiańskiego 27, 50-370 Wrocław, Poland

## Abstract

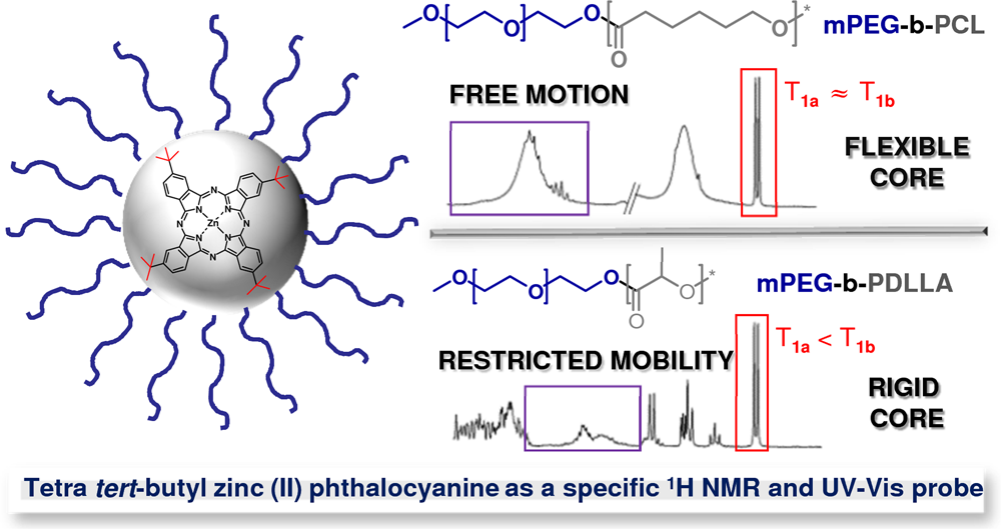

The aim of the performed studies was to thoroughly examine the internal structure of self-assembled nanocarriers (i.e., polymeric micelles—PMs) by means of a hydrophobic phthalocyanine probe in order to identify the crucial features that are required to enhance the photoactive probe stability and reactivity. PMs of hydrophilic poly(ethylene glycol) and hydrophobic poly(ε-caprolactone) (PCL) or poly(d,l-lactide) (PDLLA) were fabricated and loaded with tetra *tert*-butyl zinc(II) phthalocyanine (ZnPc-*t*-but_4_), a multifunctional spectroscopic probe with a profound ability to generate singlet oxygen upon irradiation. The presence of subdomains, comprising “rigid” and “flexible” regions, in the studied block copolymers’ micelles as well as their interactions with the probe molecules, were assessed by various high-resolution NMR measurements [e.g., through-space magnetic interactions by the 1D NOE effect, pulsed field gradient spin-echo, and spin–lattice relaxation time (*T*_1_) techniques]. The studies of the impact of the core-type microenvironment on the ZnPc-*t*-but_4_ photochemical performance also included photobleaching and reactive oxygen species measurements. ZnPc-*t*-but_4_ molecules were found to exhibit spatial proximity effects with both (PCL and PDLLA) hydrophobic polymer chains and interact with both subdomains, which are characterized by different rigidities. It was deduced that the interfaces between particular subdomains constitute an optimal host space for probe molecules, especially in the context of photochemical stability, photoactivity (i.e., for significant enhancement of singlet oxygen generation rates), and aggregation prevention. The present contribution proves that the combination of an appropriate probe, high-resolution NMR techniques, and UV–vis spectroscopy enables one to gain complex information about the subtle structure of PMs essential for their application as nanocarriers for photoactive compounds, for example, in photodynamic therapy, nanotheranostics, combination therapy, or photocatalysis, where the micelles constitute the optimal microenvironment for the desired photoreactions.

## Introduction

Polymeric micelles (PMs) are nanoscopic core/shell structures of a fairly narrow size distribution, which are formed through a molecular assembly of amphiphilic block copolymers in water. Besides the presence of two main microenvironments, outer hydrophilic and internal hydrophobic, polymeric blocks may also form different subdomains of different rigidities and have the ability to incorporate various active payloads.^1^ PMs have been successfully applied in the pharmaceutical industry for drug delivery and have shown abilities to attenuate toxicities, to enhance delivery to the desired biological sites, and to improve the therapeutic efficacy of active ingredients.^1−3^ They were found to be suitable for systemic circulation as they are large enough to prevent their rapid leakage into blood capillaries but sufficiently small enough to escape capture by macrophages in the reticuloendothelial system.^1,4^ However, the application of diblock copolymers in the fabrication of a variety of PM-based delivery systems, especially for highly insoluble bioactive agents, requires a comprehensive study of the influence of the micellar pseudophases or polymeric subdomains on the payload properties.^3,5,6^

One of the most interesting groups of porphyrin-like payloads is zinc(II) phthalocyanines (ZnPcs), which are light-sensitive molecules with exceptional stability and light absorption properties in the red/near IR region (ε > 10^5^ M^–1^ cm^–1^ for the Q band, typically located at 650–700 nm). ZnPcs not only comprise a variety of components of biosensors in bioimaging applications^7^ but are also among the most promising second generation photosensitizers (Ps) for the photodynamic therapy (PDT) of cancer.^8,9^ When illuminated with visible or infrared light, ZnPcs effectively generate highly reactive oxygen species (ROS), which cause the death of cancer cells and the tumor vasculature.^10^ The principal limitation of most ZnPc-type derivatives used as Ps is their low solubility, even in organic solvents. Moreover, the application of ZnPcs in the fabrication of a variety of PM-based delivery systems requires introducing different moieties (mostly alkyl, carboxylic acid, sulfonate, or tertiary amine) chemically attached to the peripheral aromatic rings. Consequently, in many nanocarriers, these valuable active components must be enclosed in micro- or nanosized micellar domains to maintain stability and increase their accumulation at the disease site.^11^ A variety of hydrophobic ZnPcs have recently been studied in different types of nanocarriers, including PMs of polyethyleneglycol-5000-distearoyl-phosphatidyl-ethanolamine,^12^ poly(d,l-lactic-*co*-glycolic acid) nanoparticles,^13^ mesoporous silica nanoparticles,^14^ or dimyristoyl phosphatidylcholine liposomes.^15^ One of the unique examples—tetra *tert*-butyl zinc(II) phthalocyanine of a planar structure with four *tert*-butyl peripheral groups—may act as a multifunctional probe for microenvironment structural analysis, especially for polymer-based nanocarriers and other water-borne-dispersed systems. In contrast to heavy nuclide NMR probes, requiring special pulse programs and long acquisition periods, in the simple ^1^H NMR spectrum of ZnPc-*t*-but_4_, one can see only two individual groups of signals: strong singlet at *ca.* 1.1–1.2 ppm (*tert*-butyl moieties) and multiplets at *ca.* 7.5–8 ppm, which are attributed to the aromatic ring protons. The aforementioned features make ZnPc-*t*-but_4_ an interesting probe for numerous organic microenvironments, allowing high-resolution ^1^H NMR analysis, such as the following approaches: proton relaxometry, correlation studies *via* nuclear Overhauser effect (NOE), as well as diffusometry. An added value of ZnPc-*t*-but_4_ is the presence of (theoretically) fast tumbling *tert*-butyl protons, giving only one strong signal, when their motion is not restricted—thus, the aforementioned groups are very susceptible to changing their appearance in the spectra.

Micro- and nano heterogeneous systems, found in pharmaceuticals, foods, petrochemicals, polymers, hydrogels, and organogels, can be conveniently characterized by means of various high-resolution NMR techniques.^16−18^ The most spectacular achievements of NMR self-diffusion spectroscopy applications in the field of colloids were the investigations of micellar growth and shape changes from sphere to rod-like,^19^ formation of mixed aggregates of surfactants and polymer interactions between them^20^ or biopolymers,^21^ cellulose–solvent interactions,^21^ carbon nanotube–surfactant interaction^22^ physical properties of bicelles,^23^ as well as self-organized hydrophobically functionalized polyelectrolytes.^24,25^ NMR relaxometry, that is, measurements of *T*_1_ (spin–lattice) and *T*_2_ (transverse) relaxation times, based on the principles of re-establishing the equilibrium condition after a certain pulse, is very useful for structure analysis of various materials in the liquid or solid state. Spin–lattice relaxation times describe the loss of energy, which is transferred to the surroundings in the form of heat leading to small, undetectable temperature changes—for solid-state phenomena, the aforementioned energy excess is dissipated into the surrounding lattice.^26^ The analysis of *T*_1_ relaxation is strictly connected with Bloch theory and hypothesis of the exponential recovery of spins, which is found to be very accurate for various systems; for the simplest cases, the monoexponential function may be sufficient (one population of protons with the same relaxation rates), but numerous systems, especially polymeric and/or solid ones, can be accurately fitted only to functions incorporating the occurrence of different relaxation kinetics within certain spin systems. A competing process, *T*_2_ relaxation, leading to the loss of magnetization within the *x*–*y* plane, is also measurable in a similar way (exponential recovery) and may afford additional information about certain systems, when the determination of transverse relaxation times is applicable.^26^ The aforementioned approach of NMR relaxometry is particularly useful for structure analysis of certain polymers and their blends [polycaprolactone/poly(vinyl alcohol)^27^ and starch/polycaprolactone,^28^ nanostructured inorganic (SiO_2_/TiO_2_) hybrids for drug (anti-HIV inhibitor—nevirapine) delivery applications^29^ and amorphous solid dispersions,^30^ as well as aggregates of amphiphilic poly(ethylene oxide) derivatives.^31^ For the direct investigation of the interactions between block copolymer fragments and the payload of the NOE, including basic measurements, such as 1D NOE or 2D NOESY, is a crucial analytical method.^32^ NOE methodologies have been used to study self-organization in dynamic or static systems such as PMs (including hydrophobic/amphiphilic payload loci of solubilization),^33,34^ polyelectrolyte multilayers on silica,^35^ clusters of protic ionic liquids and water,^36^ and self-assembled structures of amphiphilic star-shaped polymers.^37^

The aim of this work was to study the internal structure and the rigidity of the polymeric matrix in the micelles of amphiphilic diblock copolymers of poly(ethylene glycol) (PEG) and poly(ε-caprolactone) (PCL) or poly(d,l-lactide) (PDLLA) in view of the solubilization in their microenvironments of tetra *tert*-butyl zinc(II) phthalocyanine (ZnPc-*t*-but_4_) acting as a specific ^1^H NMR and UV–vis probe. The presence of “rigid” and “flexible” PM core-forming segments in the studied PCL and PDLLA PMs as well as their interactions with ZnPc-*t*-but_4_ were assessed by high-resolution NMR techniques: diffusion-ordered NMR (DOSY NMR), NOE, as well as *T*_1_ relaxometry. The colloidal characterization of the studied PMs was achieved from dynamic light scattering (DLS), atomic force microscopy (AFM), and DOSY NMR, while solubilization parameters of ZnPc-*t*-but_4_–PMs were extracted from the UV–vis spectra. The photochemical reactivity of ZnPc-*t*-but_4_ (i.e., photobleaching and ^1^O_2_ generation rates) was measured for its free form and for the Ps loaded in mPEG-*b*-PCL and mPEG-*b*-PDLLA micelles.

## Experimental Section

### Materials

All the reagents were used as received. Methoxypoly(ethylene glycol)-*block*-polycaprolactone [mPEG-*b*-PCL, *M*_n_ (from GPC) = 8368 Da, *M*_w_ (from GPC) = 13897 Da, and PDI = 1.66] and methoxypoly(ethylene glycol)-*block*-poly(d,l-lactide) [mPEG-*b*-PDLLA, *M*_n_ (from GPC) = 7425 Da, *M*_w_ (from GPC) = 12289 Da, and PDI = 1.65] were obtained from Akina, Inc; while tetra *tert*-buthyl zinc(II) phthalocyanine (ZnPc-*t*-but_4_), deuterium oxide (D_2_O), chloroform-*d* (CDCl_3_), and sodium trimethylsilylpropanesulfonate (DSS)—from Sigma-Aldrich. All used solvents were of reagent or analytical grade and purchased from Avantor Performance Materials. Water used in all the experiments was doubly distilled and purified by means of a Millipore (Bedford, MA) Milli-Q purification system.

### Preparation of ZnPc-*t*-but_4_-Loaded PMs by the Thin-Film Method

Micelles of mPEG-*b*-PCL and mPEG-*b*-PDLLA, loaded with ZnPc-*t*-but_4_, were prepared by a thin-film method. In the first step, appropriate amounts of the block copolymer (20 mg) and tetra *tert*-butyl zinc(II) phthalocyanine (in order to obtain the desired concentration) were dissolved in tetrahydrofuran. The obtained mixtures were placed in round-bottom flasks, followed by solvent evaporation under reduced pressure at 60 °C. The formed thin film was additionally dried at room temperature for 24 h and redissolved in deuterium oxide (3 mL) during stirring at 60 °C for 30 min, followed by sonication (60 °C, 30 min), and slowly cooled to room temperature. The obtained PM solutions were filtered through syringe filters (0.22 μm). The concentration of the block copolymer (6.7 mg/mL) was around one order of magnitude higher in comparison to phthalocyanine (around 0.1 mg/mL) in order to meet requirements of high-resolution NMR techniques. Generally, the concentration of the solubilized compound should be appropriate for ^1^H NMR analysis: too low concentration results in unacceptable acquisition times, while very high concentration may lead to a very low spectra resolution.^26^ A higher concentration of ZnPc-*t*-but_4_ is not possible (overloading of PMs), while their lower values will be too low for accurate high-resolution NMR analysis. Characterization of PCL and PDLLA micelles are as follows. The size distribution (i.e., the hydrodynamic diameter, *D*_H_) and polydispersity index (PDI) of all PMs were determined by DLS using a Zetasizer NanoZS Instrument (ZEM4228, Malvern Instruments, UK) equipped with a 4 mW He–Ne laser (λ = 633 nm) and with noninvasive backscattering detection at a scattering angle of 173° in optically homogeneous polystyrene microcuvettes. The autocorrelation function was converted in a number-mean hydrodynamic radius distribution with the Dispersion Technology Software 8.10 from Malvern Instruments. Each measurement was repeated at least 3 times, and the average result was accepted as the final hydrodynamic diameter (*D*_H_) with a standard deviation, whenever all the values fluctuated within a reasonable experimental error. The morphology and dimensions of the PMs were examined by AFM using a Veeco NanoScope Dimension V atomic force microscope with an RT ESP Veeco tube scanner (Plainview, New York, United States). The scanning speed was 0.5 Hz, and a low-resonance frequency pyramidal silicon cantilever resonating at 250–331 kHz was employed (at a constant force of 20–80 N/m). The amplitude of the resonance was set manually to the lowest possible value for stable imaging within the contamination layer present on the surface. Before observations, the PM solutions (diluted 15-fold in double-distilled water) were placed on a cover glass surface and allowed to dry at room temperature. Then, excess micelles were removed by rinsing the surfaces with double-distilled water for 30 min and drying at room temperature.

### NMR Techniques and Methodologies

All NMR experiments were conducted on a Bruker AMX600 instrument in deuterium oxide (D_2_O, 99.9% at. D) or chloroform-*d* (CDCl_3_, 99.9% at. D) at a stabilized temperature of 298 K. In ^1^H NMR spectra, chemical shifts were referenced to the TSP signal as an external standard (in case of D_2_O) or DSS as an internal standard (in case of CDCl_3_) with a spectral resolution of 0.055 Hz. Concentrations of ZnPc-*t*-but_4_ in analyzed micelle systems are listed in Table 2, while the block copolymer concentration (67 mg/mL) is constant for all samples. Spin–lattice relaxation (*T*_1_) measurements were conducted using the inversion-recovery sequence (*t1ir* pulse program from Bruker repository). Intensities for 20 increments of recovery times were used for each measurement. Relaxation times for protons in both the hydrophobic and the hydrophilic segments, mPEG-*b*-PCL and mPEG-*b*-PDLLA micelles, were estimated by fitting to an exponential model, as described by eq 1
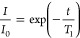
1where *t* is the recovery time and *I*_0_ is the intensity immediately after the 180× pulse.

Similarly, spin–lattice relaxation times for *tert*-butyl protons (δ = 1.175–1.186 ppm) of ZnPc-*t*-but_4_ in mPEG-*b*-PCL and mPEG-*b*-PDLLA micelles were determined, although the obtained data were fitted to a monoexponential function (see eq 1) as well as a biexponential model, assuming the coexistence of the two populations of protons with different *T*_1_ values (*T*_1a_ and *T*_1b_). DOSY NMR measurements were performed for empty and loaded with ZnPc-*t*-but_4_ mPEG-*b*-PCL and mPEG-*b*-PDLLA micelles utilizing the *dstebpgp3s* Bruker pulse program. The gradient amplitude γ and diffusion time Δ were constant and equal to 2.675 108 Hz/T and 493 ms (for PMs) or 197.4 ms (for ZnPc-*t*-but_4_ diffusion coefficient determination in both systems), respectively. The maximum (initial) gradient strength *G* was set at 32.35 T/m, while gradient duration δ was at 4.8 ms (for PMs) or 1.5 ms (for ZnPc-*t*-but_4_ diffusion coefficient determination in both systems). Peak areas (PEG peak at 3.718 ppm for PMs and *t*-but peak at 1.175–1.186 ppm for ZnPc-*t*-but_4_) for 16 increments of different gradient strengths, decreasing to about 5–20% of its initial value, were used for each diffusion coefficient measurement. Appropriate mono-, bi-, and triexponential functions, according to eq 2
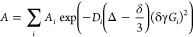
2were fitted to the data, where the total intensity (*A*) is a weighted sum of individual contributions (*A*_*i*_) and diffusion coefficients (*D*_*i*_) of differently diffusing populations. The particular equations for mono-, bi-, and triexponential functions are presented in Supporting Information, together with the calculated values of the diffusion coefficients (see Table S6). Number-weighted PMs and (macro)molecules’ hydrodynamic radii were calculated using the Stokes–Einstein equation (eq 3)
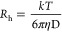
3where *R*_h_ is the hydrodynamic radius, *k* is the Boltzmann constant (=1.38 × 10^–23^ m^2^ kg s^–2^ K^–1^), *T* is the absolute temperature (=298 K), η is the viscosity for D_2_O (1.09 mPa s at 298 K), and *D* is the diffusion coefficient determined above. 1D selective NOE experiments (1D NOE NMR) were performed for mPEG-*b*-PCL and mPEG-*b*-PDLLA micelles, loaded with ZnPc-*t*-but_4_, utilizing a *selnogp* Bruker pulse program. The mixing time was equal to the *T*_1_ relaxation time of the corresponding protons (502 ms for methylene protons at 4.037 ppm in mPEG-*b*-PCL and 2.906 s for methyl protons at 1.598 ppm in mPEG-*b*-PDLLA). The relaxation delay and acquisition time were set at 3 and 2.73 s, respectively.

### UV–vis Measurements

The UV–vis spectra were recorded from 200 to 800 nm on a U-2900 spectrophotometer (Hitachi, Japan) using a sampling interval of 1.0 and a scan rate of 400 nm/min. For the determination of tetra *tert*-butyl zinc(II) phthalocyanine [ZnPc-*t*-but_4_], concentrated samples were diluted with tetrahydrofuran, a water-miscible organic solvent that can molecularly dissolve the analyzed Ps, to obtain THF/water 5:1 mixtures. Spectra of aqueous dispersions of mPEG-*b*-PCL and mPEG-*b*-PDLLA micelles, empty and loaded with ZnPc-*t*-but_4_, were also collected. All measurements were carried out in synthetic quartz glass cuvettes (optical path length 10 mm) using 1.5 mL of the solution volume. The detailed methodology for UV–vis measurements and calculations is described in Supporting Information (subsection 4—solubilization of ZnPc-*t*-but_4_ in mPEG-*b*-PCL and mPEG-*b*-PDLLA micelles). The compatibility between polymers and those incorporated in their matrix payloads, including biologically active compounds and molecular probes, may be described by the solubility and miscibility parameters.^38^ The calculation of solubility (δ_*x*_) and miscibility (Flory–Huggins interaction parameter—χ) parameters was performed for all polymer blocks (PEG, PCL, and PDLLA) and the probe—tetra *tert*-butyl zinc(II) phthalocyanine in order to determine the compatibility of the payload and polymeric matrices. For the detailed methodology and results, see the Supporting Information (subsection 5—solubility and miscibility parameters for ZnPc-*t*-but_4_ in mPEG-*b*-PCL and mPEG-*b*-PDLLA micelles).

### Photobleaching and ^1^O_2_ Generation of ZnPc-*t*-but_4_-Loaded PMs

Photokinetic studies of photobleaching and ^1^O_2_ generation in micro-heterogeneous systems were performed in open quartz cuvettes (1 cm optical path) with continuous stirring using an OPTEL fiber illuminator (Opole, Poland) with a long-pass glass filter (Schott Glaswerke GmbH, Mainz, Germany) to separate the 600–750 nm spectral interval. The photobleaching rate was evaluated spectrophotometrically by monitoring the ZnPc-*t*-but_4_ absorption spectrum in the range of 300–800 nm upon exposure to the lamp working at a 100 mW/cm^2^ fluence for 3 mL of solutions containing the free or encapsulated ZnPc form in FA-functionalized PMs (system 4 in Table 1). Measurements for free ZnPc-*t*-but_4_ were conducted in the 1% PEG water solution to prevent the aggregation and loss of absorbance.^34^ In the photobleaching measurements, the solution concentrations were selected in order to obtain an initial ZnPc-*t*-but_4_ main Q-band peak (676 nm) absorbance of less than 1.0. For both free and encapsulated ZnPc-*t*-but_4_, the measurements were performed at approximately 10 different irradiation time intervals. The rate of ^1^O_2_ generation was measured in the same manner as photobleaching, that is in the presence of 0.14 mM 9,10-anthracenediyl-bis(methylene)dimalonic acid sodium salt (ABMDMA). The concentration of ZnPc-*t*-but_4_ was 5 μM in both the free and PM-encapsulated forms. The singlet oxygen generation rate constant (*k*_v_) was calculated from the ratio of ABMDMA absorbance at 400 nm before and after irradiation as a function of the irradiation time according to Lamch et al*.*^4^ The photobleaching and ^1^O generation measurement results are presented in Figure 4.

**Table 1:**
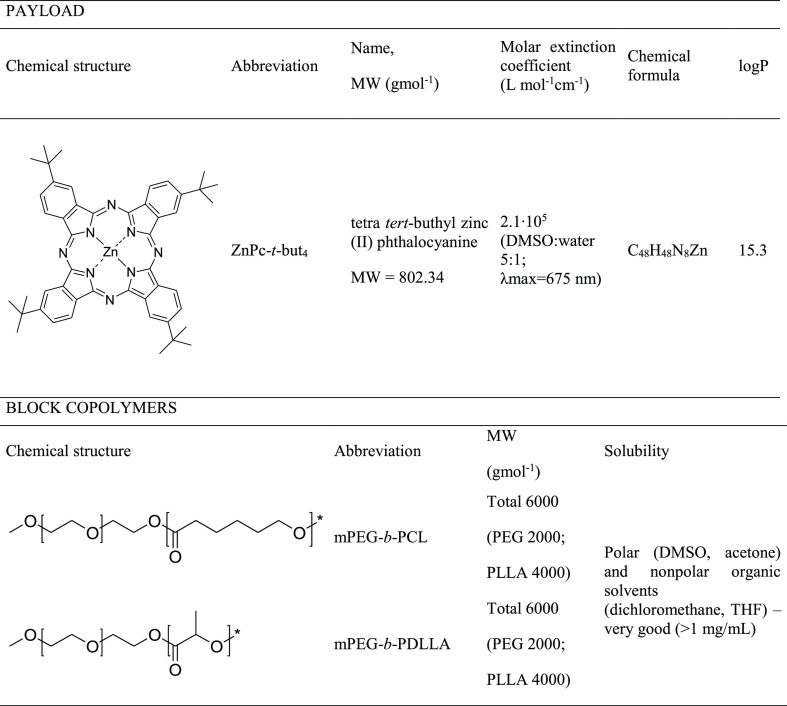
Structures and Properties of the Studied Zinc Phthalocyanine and Block Copolymers

## Results and Discussion

### Preparation and Physicochemical Characterization of PMs

PCL and PDLLA PMs, empty and loaded with zinc phthalocyanine (ZnPc-*t*-but_4_), were prepared *via* the thin-film method with the use of mPEG-*b*-PCL and mPEG-*b*-PDLLA block copolymers (detailed composition and properties of the nanosystems are presented in Tables S1 and S3). The resulting micellar solutions were transparent, with no signs of block copolymer precipitation or PM aggregation. The results obtained by DLS (see original autocorrelation functions and number-mean statistic bars in Figures S1 and S2) demonstrate that the PMs had average hydrodynamic diameters (*D*_H_) of about 40 nm (mPEG-*b*-PCL micelles) and 25 nm (mPEG-*b*-PDLLA micelles) with relatively low polydispersity indices (PDI between 0.09 and 0.27 for both systems). The influence of ZnPc-*t*-but_4_ encapsulation on the values of the mean hydrodynamic diameter of PMs, *D*_H_, is mostly connected with the packing of the polymer chains inside the micelles’ cores, due to the presence of highly hydrophobic molecules at *ca.* 10–15 wt %. On the other hand, the polydispersity of PMs’ dimensions typically provides an accuracy of *ca*. 10–20% of the mean value. The aforementioned reasons make it difficult to observe the slight influence of the payload molecules on PM diameters.^1,3,38^ Therefore, it is worth noticing that the influence of ZnPc-*t*-but_4_ addition on the mean value of micelles *D*_H_ was found to be very slight and fluctuating within a reasonable measurement error (*ca.* 1–2 nm in comparison to hydrodynamic diameters around 25–40 nm).

**Figure 1:**
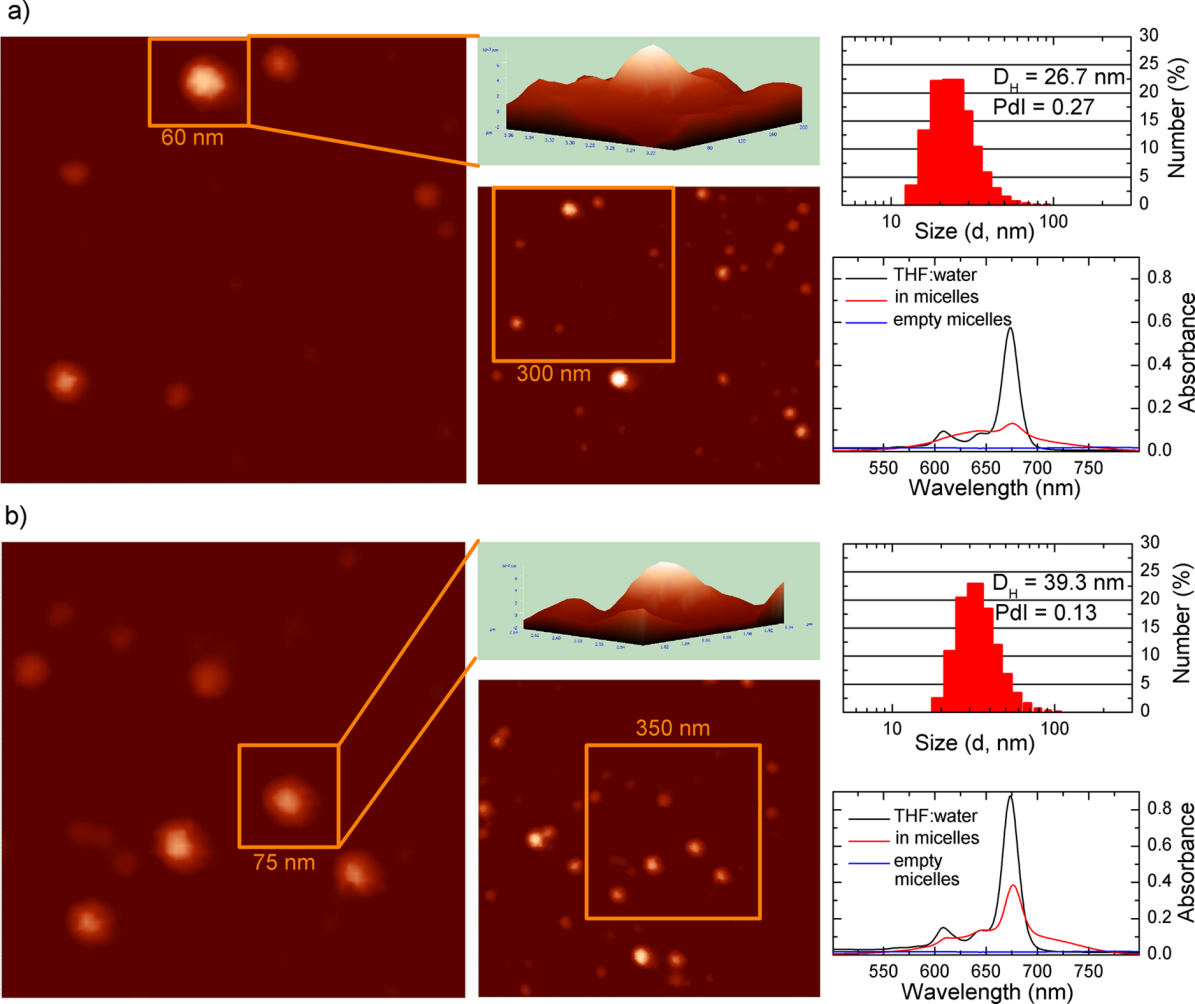
AFM images, DLS results, and UV–vis spectra of *tert*-butyl zinc(II) phthalocyanine-loaded mPEG-*b*-PDLLA (a) and mPEG-*b*-PCL (b) micelles. The legend for UV–vis spectra: ZnPc-*t*-but_4_-loaded PMs dissolved in the THF/water (5:1, v/v) mixture—black line, ZnPc-*t*-but_4_-loaded PMs in aqueous solution—red line, and empty PMs—blue line.

The usefulness of ZnPc-*t*-but_4_ as an appropriate spectroscopic probe molecule for studying mPEG-*b*-PCL and mPEG-*b*-PDLLA micelle systems was proved by UV–vis measurements and described by the percentage loading efficiency and the percentage Ps/polymer ratio as well as the PM–water partition coefficient. The aforementioned parameters exhibited an appropriate equilibrium between the probe entrapped within the hydrophobic micelle core and simply dissolved in water, indicating sufficient hydrophobicity of the used ZnPc-*t*-but_4_ phthalocyanine. The loading efficiency for both analyzed systems was approximately 100%. The maximal ZnPc-*t*-but_4_ concentrations were 102.8 μM (in mPEG-*b*-PCL micelles) and 67.4 μM (in mPEG-*b*-PDLLA micelles), with the corresponding percentage Ps/polymer ratios of 15.3 and 10.0%, respectively. Using the max[ZnPc-*t*-but_4_]_micelle_ and [ZnPc-*t*-but_4_]_aqueous_ values, the partition coefficients of ZnPc-*t*-but_4_ were calculated (Table S3). The higher maximal ZnPc-*t*-but_4_ concentration and percentage Ps/polymer ratio (*ca.* 15% in contrast to *ca.* 10%) were obtained for the Ps loaded in mPEG-*b*-PCL than in mPEG-*b*-PDLLA micelles. The observed phenomenon can be caused by the enhanced loading capacity of flexible subdomains in the PCL matrix in comparison to the PDLLA microenvironment with partially restricted mobility regions. The differences between the degree of hydrophobicity of both block copolymers probably played no role, due to nearly the same (*ca.* 15) PM–water partition (log *P*) coefficients for both copolymers. The determined solubilization parameters are in good agreement with similar nanosystems (ZnPc or their derivatives in mPEG-*b*-PLLA micelles), despite the fact that the percentage Ps/polymer ratio is about one order of magnitude higher in mPEG-*b*-PCL and mPEG-*b*-PDLLA than in mPEG-*b*-PLLA micelles.^34^ THF was chosen as a solvent for the ZnPc-*t*-but_4_ concentration determination because both block copolymers and photoactive probe ZnPc-*t*-but_4_ were in the fully dissolved monomeric (nonaggregated) form in this solvent. The obtained UV–vis (Figure 1a,b) spectra of the PM systems indicated that ZnPc-*t*-but_4_ was solubilized in a nonaggregated form, as proved by the presence of the Q-band at about 675 nm in all the systems (solution in THF, ZnPc-*t*-but_4_-loaded mPEG-*b*-PCL, and mPEG-*b*-PDLLA micelles). The very low ZnPc-*t*-but_4_ aqueous solubility—2.45 × 10^–14^ mg/mL—a few orders of magnitude lower than its detection limits in the solution via UV–vis spectroscopy makes it very difficult to study in its nonsolubilized form.^8,11,12^ Generally, the phthalocyanine molecules are mostly solubilized within the hydrophobic domains of PMs. The aforementioned phenomenon is strictly connected with a high tendency of the planar structures—like phthalocyanines—to aggregate in polar solvents, followed by the loss of photoactivity and precipitation of the aggregates.^13,15,34^ Unfortunately, differential scanning calorimetry (DSC) measurements for PMs are not possible—the subtle structure of subdomains in such systems is hardly visible on DSC curves. Moreover, the studies of PMs should be performed in aqueous systems, where numerous phenomena (e.g., connected with the hydration of the PEG chain) affect DSC curves, making it very difficult to gain valuable information.^5,27^ The solubility (δ_p_) of particular block copolymer fragments as well as the miscibility parameters (χ) between them may help in predicting such compounds’ performance in aqueous systems, especially for a nonionic system.^38^ On the other hand, the formation of hydrogen bonds, especially by the hydrophilic parts, could make those considerations pointless, due to the inconclusive actual structure of corona-forming blocks, composed of the polymer and hydration water molecules. Generally, the minimization of the miscibility parameter (χ) between compounds indicates a more probable formation of the solution/molecular blend. The calculated values of the miscibility parameters of particular blocks (hydrophilic PEG as well as hydrophobic PCL and PDLLA, see Table S4) in water indicate that the most preferable is the formation of the corona-core structure with the internal PCL or PDLLA block and external PEG fragment, extending into aqueous solution. In order to show the compatibility between the active payload—tetra *tert*-butyl zinc(II) phthalocyanine and appropriate microenvironments—core-forming PCL or PDLLA and hydrophilic, water miscible PEG—appropriate miscibility (χ) parameters were calculated (see Table S5). The obtained values indicate that ZnPc-*t*-but_4_ is nearly ideally miscible with PDLLA and PCL blocks (χ parameter value lower than 1) in contrast to PEG (χ parameter value higher than 2—possible partial miscibility or miscibility under certain conditions). The obtained results are in good agreement with the logP values of ZnPc-*t*-but_4_ in PCL and PDLLA (15.3 and 15.2, respectively; see Table S3), showing that the studied phthalocyanine preferably accumulates in the hydrophobic microdomains rather than in the hydrophilic ones. Moreover, our solubility and miscibility parameter investigations confirm the solubilization locus of ZnPc-*t*-but_4_, which was investigated by high-resolution NMR studies, within the core of PMs, which is discussed in detail in the following paragraphs.

### General NMR Studies of PMs

Studies of mPEG-*b*-PCL and mPEG-*b*-PDLLA in CDCl_3_ and D_2_O provided evidence of the micelle formation (Table 2). In CDCl_3_, which is a nonselective solvent for both block copolymers, the complete structural resolution of the whole macromolecules was observed. PCL methylene as well as PDLLA methyl and methine protons together with the PEG protons were completely resolved, indicating that both the diblock copolymers were homogeneously dissolved as a solution of nonaggregated macromolecules. A similar effect is observed for the corona-core structure in mPEG-*b*-PCL and mPEG-*b*-PDLLA micelles in D_2_O in spite of the fact that particular blocks are situated in different microenvironments, that is, internal hydrophobic (PCL and PDLLA) and outer aqueous corona (PEG). The internal core acts as the host domain for the hydrophobic probe, stabilizing its photochemical properties, mostly via significantly reduced unwanted aggregation rates. The hydrophilic blocks interact favorably with the water molecules through the formation of hydrogen bonds to create an exterior hydrophilic corona that extends into aqueous media and stabilizes the structure of the polymeric micelle.^6,39,40^

**Table 2:**
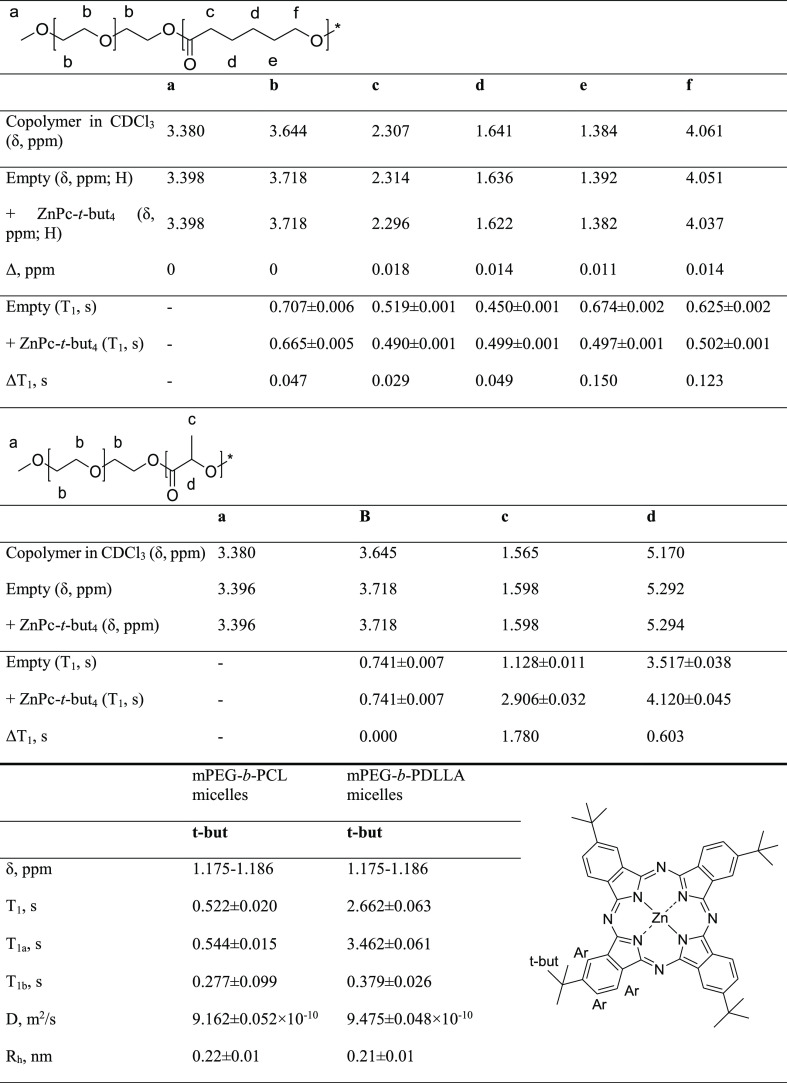
^1^H NMR Chemical Shifts and Spin–Lattice Relaxation (*T*_1_) Measurements for Empty and ZnPc-*t*-but_4_ mPEG-*b*-PCL-Loaded PMs and mPEG-*b*-PDLLA Micelles

The chemical shifts of both block copolymers as well as empty and ZnPc-*t*-but_4_-loaded PMs are calculated from the NMR spectra and are presented in Table 2. According to the measurements (ZnPc-*t*-but_4_-loaded PMs in D_2_O), *tert*-butyl protons were observed in the spectra of both nanosystems (two sharp signals with chemical shift values of 1.175 and 1.186 ppm) (Table 2 and Figure 3). In the spectra of ZnPc-*t*-but_4_-loaded PMs, D_2_O signals (multiplets), attributed to the aromatic protons in phthalocyanine rings, were also visible at 7.7–7.8 ppm. This observation indicates the presence of ZnPc-*t*-but_4_ molecules in the analyzed solution, most probably as solubilized within PM cores due to their unambiguous hydrophobicity. The concentration of free, nonsolubilized in PM ZnPc-*t*-but_4_ molecules is equal to its aqueous solubility (2.45 × 10^–14^ mg/mL), thus no ^1^H NMR signals, contributing to their presence in the solution, may be observed.^26^ According to the probe structure, all *tert*-butyl protons should be observed in the spectra as only the singlet signal. The presence of two signals in the spectra of ZnPc-*t*-but_4_-loaded mPEG-*b*-PCL and mPEG-*b*-PDLLA micelles may suggest a somewhat limited mobility of ZnPc-*t*-but_4_ molecules.^26^ More detailed information about the interactions between block copolymers and ZnPc-*t*-but_4_ was obtained from the analysis of mPEG-*b*-PCL and mPEG-*b*-PDLLA chemical shifts. The chemical shifts of specific peaks in the NMR spectra suggest that the local environment surrounding the corresponding hydrogen atom has changed, presumably due to the presence of the solute near that hydrogen. For all methylene protons in the PCL chain of mPEG-*b*-PCL micelles, significant differences in the value of the chemical shifts were observed between ZnPc-*t*-but_4_-loaded and empty micelles. The presence of the aromatic ring provides a negative contribution to the local magnetic field, leading to low-frequency shifts of the neighboring macromolecules’ protons.^39^ Such a phenomenon, that is, significant differences in the value of the chemical shifts between ZnPc-*t*-but_4_-loaded and empty micelles, was not observed for mPEG-*b*-PDLLA.

### Studies on the Rigidity of PM Core-Forming Segments via NMR Diffusometry and Relaxometry

Polymeric supramolecular aggregates in the form of PMs often feature a range of microscopic environments, each of which having distinct values of the NMR observables. Crucial information about nano- and micro-heterogeneous systems, as well as solubilized in their microenvironment active payloads, may be assessed by *T*_1_ (longitudinal or spin–lattice) and/or *T*_2_ (transverse) relaxation time measurements. The spin–lattice relaxation (*T*_1_) characterizes the rate at which the longitudinal *M*_*z*_ component of the magnetization vector is re-established exponentially toward its equilibrium value of magnetization, *M*_*z*_(0), thus reaching a thermodynamic equilibrium with its surroundings (the “lattice”). The spin–spin relaxation (*T*_2_) is caused by the energy exchange around the nuclei without a loss of energy to the surrounding lattice, resulting in the decay of transverse (*M*_*x*–*y*_) magnetization to *M*_*x*–*y*_(0).^26^ The signal attenuation is related to the underlying probability distribution of exponential components through a Laplace transformation and may be represented by the general form exp(−*t*Γ), in which *t* is a weighting amplitude and Γ is a decay rate constant. The decay rate is dependent on the spin–lattice relaxation time (*T*_1_) for the spin-echo block attenuation or self-diffusion coefficient (*D*) in the case where the studied molecular or supramolecular species experience free diffusion. Spin–lattice relaxation time measurements provide useful information about the polymer segment’s local mobility, while diffusion-ordered NMR reports about mean values of macromolecules and aggregated hydrodynamic diameter.

In the field of dispersed systems, *T*_1_ and/or *T*_2_ relaxation time measurements of macromolecules and colloids have been employed to investigate the motion of the associated water in poly(styrene sulfonate) and poly(allylamine) hydrochloride multilayers,^35^ the dependence of proton *T*_2_ relaxation times of particular fragments (amino acid residues) in gelatin on the ratio of κ-carrageenan to gelatin,^41^ the dispersion state of cellulose nanocrystals [in the presence of carboxymethylcellulose and poly(ethylene oxide) polymers],^42^ as well as the structure, shape, and dynamics of nonionic, PEG-ylated surfactant micelles.^31^ Most often, the aforementioned studies involved the analysis of only water molecules, enabling to gain information about the solvated or hydrated domains. On the other hand, such an approach is available only for hydrophilic/amphiphilic microenvironments such as the outer corona or the interior of liposomes/polymersomes but not for the hydrophobic domains with limited mobility (like non-water soluble polymers or their derivatives). Generally, for small (with a molecular weight of up to about few hundred Daltons) rapidly tumbling molecules, the longitudinal and transverse relaxation rates are identical (*T*_1_ = *T*_2_). Moreover, slow relaxation (i.e., high values of *T*_1_) is a characteristic for small molecules in low viscosity solvents (in the so-called extreme narrowing limit). Typically, for low-molecular weight compounds, *T*_1_ relaxation times of protons are around 1 s but may be as short as around 10^–1^ to 10^–2^ s (100–10 ms) for some low-molecular weight polymers in both the crystalline or amorphous phase.^29^ Thus, when the tumbling rate of a molecule is limited, due to its high-molecular weight, increased solvent viscosity, or reduced sample temperature, the transverse relaxation times are shorter in comparison to the longitudinal ones.^26,43^ In contrast to the transverse (*T*_2_) relaxation time, the spin–lattice relaxation time (*T*_1_), as a function of the correlation time, at first decreases, then goes through minimum, and finally increases. *T*_1_ relaxation times, depending mostly on the molecular weight, type of solvent, and other factors limiting mobility (high viscosity and low temperature), may indicate how the motion of molecules/their fragments is restricted. Generally, molecules bound via hydrogen bonds, placed in nano- or microdomains or highly viscous microenvironments, are characterized by different, that is, higher or lower, according to their molecular weight, values of *T*_1_ relaxation times in comparison to their analogues with high mobility. Spin–lattice relaxation times are also indirectly connected with the viscosity of a solvent/environment due to the reduced tumbling rate, resulting in the reduced *T*_1_ values.^26^ For some high-molecular weight polymers, the opposite performance may be observed—*T*_1_ relaxation times lengthen with the increase of structure rigidity.^29^ On the other hand, *T*_2_ relaxation times for some, especially high-molecular weight compounds such as polymer chains, are difficult to be determined (e.g., each fragment of the macromolecule may have its own value of *T*_2_) and thus are not applicable in such system investigations.^26^ In order to determine the mobility of protons in subdomains and microphases of PCL and PDLLA, the following experiments were conducted: *T*_1_ relaxation times for each measurable (i.e., possessing sufficient intensity) proton groups in mPEG-*b*-PCL and mPEG-*b*-PDLLA micelles (empty and loaded with ZnPc-*t*-but_4_) were determined as well as spin–lattice relaxation times for *tert*-butyl protons of ZnPc-*t*-but_4_ in PM systems. The obtained data points for *tert*-butyl protons of ZnPc-*t*-but_4_, entrapped within appropriate PMs, were fitted to the mono- and biexponential functions (i.e., assuming the presence of one or two different *T*_1_ relaxation times values)—see Table 2. On the other hand, the monoexponential fitting was sufficient (biexponential fitting yielded two equal values of *T*_1_ relaxation times) for protons in the given PCL, PDLLA, and PEG blocks. Thus, two different values of spin–lattice relaxation times (*T*_1a_ and *T*_1b_) for one signal (group of protons) were applicable only for *tert*-butyl protons in the probe phthalocyanine molecule. As it was stated earlier, PCL is a semicrystalline polymer, although only an amorphous phase (especially “interphase” between the rigid and flexible regions) may act as the host space for the probe molecule, while PDLLA is an amorphous, glassy polymer. Our *T*_1_ investigations were specially focused on the rigidity and flexibility subdomains in the amorphous phase, due to the possibility of probing with ZnPc-*t*-but_4_.

**Figure 2:**
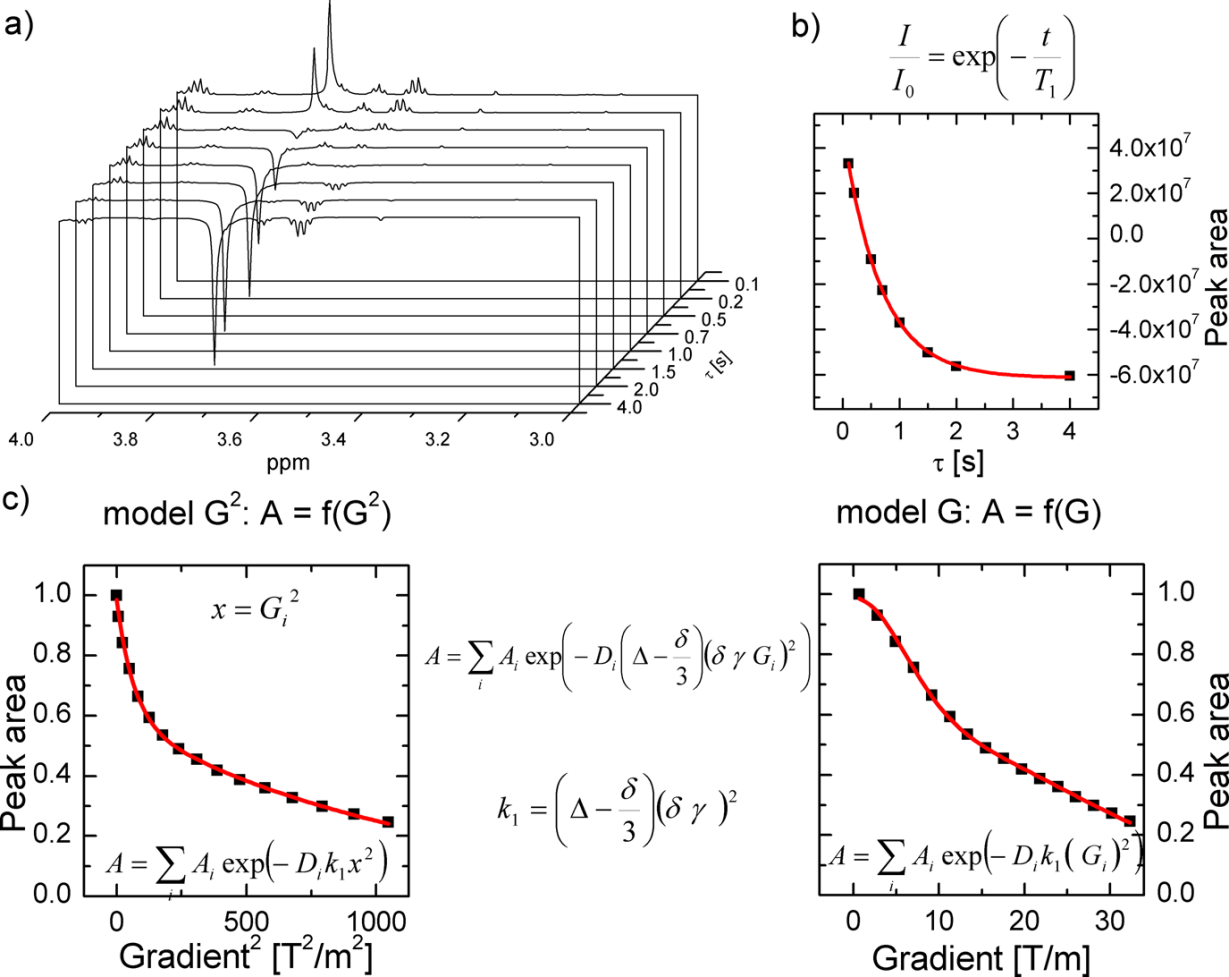
Representative graphs of 3D NMR spectra for *T*_1_ relaxation times and diffusion coefficient calculation (upper left panel a), determination of *T*_1_ relaxation times, exemplified for PEG peaks of ZnPc-*t*-but_4_-loaded mPEG-*b*-PCL micelles (right panel b), and the applied models for diffusion coefficient calculation (PEG peaks of ZnPc-*t*-but_4_-loaded mPEG-*b*-PCL micelles as an example) (lower left panel c).

The *T*_1_ relaxation times at 25 °C were measured for protons in both the hydrophilic and the hydrophobic components of empty and ZnPc-*t*-but_4_-loaded mPEG-*b*-PCL and mPEG-*b*-PDLLA micelles as well as *tert*-butyl protons of the solubilized phthalocyanine (Table 2 and representative Figure 2a,b). In mPEG-*b*-PCL micelles, *T*_1_ times varied from 0.450 s (protons d at 1.636 ppm in the empty system) to 0.707 s (PEG protons b at 3.718 ppm). Generally, the aforementioned results are consistent with the structure of the PCL PM core (semicrystalline, degree of crystallinity dependent on the temperature, mean molecular weight, as well as the value of the critical micelle concentration)—a highly ordered, dense polymeric matrix (ρ = 1.20 g/mL) allows fast spin recovery due to energy dissipation by the neighboring molecule fragments.^5,43^ The most significant difference (Δ*T*_1_ > 0.100 s) between the empty and loaded with ZnPc-*t*-but_4_ PMs was observed for e and f protons (Table 2), indicating preferable interactions between particular polymer moieties and phthalocyanine peripheral rings with the attached *tert*-butyl groups. In mPEG-*b*-PDLLA micelles, the spin–lattice relaxation time for protons b in the PEG chain (3.718 ppm) was equal to 0.741 s (the same value for empty and ZnPc-*t*-but_4_-loaded PMs). The obtained results (*T*_1_ values around 0.700 s for protons in hydrophilic fragments) indicated that PEG chains in all the studied PMs have similar mobility consistent with the corona-core model with the PM corona extending into the aqueous media—typical values for solvent-borne systems. The *T*_1_ times of hydrophobic protons in mPEG-*b*-PDLLA micelles (Table 2) for empty systems (c signals—1.128 s and d—3.517 s) were shorter than for the ZnPc-*t*-but_4_-loaded ones (c signals—2.906 s and d—4.120 s). The *T*_1_ relaxation times for hydrophobic protons in ZnPc-*t*-but_4_-loaded PMs (methylene protons e and f in mPEG-*b*-PCL and methyl c and methine d in mPEG-*b*-PDLLA) were significantly longer in comparison to PMs with no solubilized Ps. Long *T*_1_ relaxation time values for relatively low-molecular weight polymers are consistent with the amorphous structure with randomly situated chemical moieties, enabling slower energy dissipation in comparison to highly ordered, rigid structures.^26^ The aforementioned facts are in good agreement with the amorphous PDLLA micellar core structure.

*T*_1_ relaxation times of ZnPc-*t*-but_4_, as the NMR probe for careful studies of PCL and PDLLA domains in PMs, were investigated via fitting to the mono- and biexponential functions. The aforementioned approach^5,27,29^ constitutes a powerful tool for systems comprising regions of different rigidities, especially semicrystalline and amorphous polymers in which “flexible” and “rigid” subdomains may coexist within one microphase. Although the term “rigid-flexible” polymers most often refers to biopolymers like proteins, it may be also used to describe the structure of micro-heterogeneous systems like self-assembled synthetic block copolymers.^27−31^ The aforementioned subdomains are hardly detectable via conventional methods as X-ray diffraction or DSC but may be thoroughly studied by means of combined probing and high-resolution NMR. Generally, the miscibility of polymers as well as the entrapment of low-molecular weight molecules (e.g., drugs and chemical probes) in a polymeric matrix is more possible in the amorphous part, especially the flexible one, because co-crystallization, although probable, is uncommon.^27^ For planar phthalocyanines, the solubilization within borders between the polymeric subdomains is preferable, so some part of the molecule may experience different local environments in contrast to the other one. Spin–lattice relaxation times (*T*_1_) of *tert*-butyl protons in ZnPc-*t*-but_4_, obtained via fitting to the monoexponential functions, were equal to 0.522 s (ZnPc-*t*-but_4_-loaded mPEG-*b*-PCL micelles) and 2.662 s (ZnPc-*t*-but_4_-loaded mPEG-*b*-PDLLA micelles). Fitting to the biexponential function gave a higher *R*^2^ (almost equal to 1), better than the fitting to the monoexponential one, showing that the presence of two different values of *T*_1_ relaxation times for such spin systems is quite possible. Generally, the protons may experience two different *T*_1_ relaxation times: slower (denoted *T*_1a_) and faster (denoted *T*_1b_). In PEG-*b*-PCL micelles, the aforementioned spin–lattice relaxation times were equal to 0.544 and 0.277 s, respectively, indicating the accumulation of ZnPc-*t*-but_4_ molecules at the boundary between the two subdomains of different flexibilities. The difference between both relaxation times is relatively small, thus the motion of the phthalocyanine molecules is only partially restricted. The most possible mechanism involves the formation of a flexible amorphous phase with solubilized phthalocyanine molecules. Two values of relaxation times for the same spin system indicate that the aforementioned chemical groups are placed in different environments. The different environments may comprise two regions of different flexibilities or their interface (some parts of the molecule interact with one subdomain, while the other fragment—with the second one).^16−18,21^ Generally, the accumulation of phthalocyanine molecules is more probable at the interface due to its planar structure, although ZnPc-*t*-but_4_ may be also present in the interiors of the aforementioned subdomains.^15−17^ Thus, in PEG-*b*-PDLLA micelles, the *T*_1a_ and *T*_1b_ relaxation times were 3.462 and 0.379 s, while their relative strengths (contributions) were equal to 3.64 × 10^6^ and 9.68 × 10^5^, respectively. The difference between *T*_1a_ and *T*_1b_ relaxation times is about 3 s, showing that ZnPc-*t*-but_4_ molecules are accumulated within two different subdomains of different flexibilities or, possibly, at their interface due to their planar structure with a high affinity to energetically unsaturated surfaces. The relative strength (contribution) of each *T*_1_ population was determined as *A*_1a_ and *A*_1b_ for *T*_1a_ and *T*_1b_, respectively (see eq 1). All the above-mentioned data are listed in Table 2. The structure of the PDLLA core—amorphous polymer—indicates the coexistence of at least two subdomains: rigid amorphous and flexible amorphous. The aforementioned results are likely connected with the differences between the microenvironments of the PCL and PDLLA cores, studied widely by ^1^H NMR and UV–vis techniques. More restricted mobility of ZnPc-*t*-but_4_ in the PDLLA core is consistent with the spectral changes of phthalocyanine, visible in UV–vis spectra (Q-band is much broader, possibly due to slower absorption/emission photoprocesses, see Figure 1b). In PCL micelle cores, loaded with ZnPc-*t*-but_4_, the aforementioned effect is hardly visible, due to the higher mobility of ZnPc-*t*-but_4_ in flexible amorphous subdomains. In a highly ordered PCL core, *tert*-butyl protons possess neighbors in the polymer structure, enabling fast recovery due to the longitudinal mechanism, in contrast to lower rigidity in an amorphous PDLLA domain, requiring a longer time to obtain full energy dissipation. The whole relaxometry investigations were consistent with the results obtained from the UV–vis spectroscopy experiments and structural analysis by ^1^H NMR, which indicated the limited mobility in ZnPc-*t*-but_4_-loaded polymeric micelle cores as well as differences between the mobility of protons in mPEG-*b*-PCL and mPEG-*b*-PDLLA micelles.

The NMR diffusometry experiments (DOSY NMR), performed at 298 K, were carried out for protons in the hydrophilic PEG chains of empty and ZnPc-*t*-but_4_-loaded mPEG-*b*-PCL and mPEG-*b*-PDLLA micelles as well as *tert*-butyl protons of the solubilized phthalocyanine (see representative graphs in Figure 2a,c). The values of self-diffusion coefficients, obtained through fitting the signal attenuation data for two diffusing populations, are presented in Table 3 (ZnPc-*t*-but_4_-loaded PMs) and Table S1 (empty PMs). Excellent fits to the model were obtained as judged by the *R*^2^ (adjusted root mean square) values close to 1 (see fitting to mono-, bi-, and triexponential G^1^ and G^2^ models, as listed in Table S6; the most accurate results are marked bold). This fact also indicates that no other species (aggregated or cross-linked micelles, chemically cleaved polymers, *etc.*) are present in the studied solutions.^16,18^ The diffusion coefficients for the two populations were in the 10^–10^ (nonaggregated block copolymer) and 10^–11^ m^2^/s (PMs) range. Number-weighted particle hydrodynamic diameters were calculated using the Stokes–Einstein equation using the self-diffusion coefficients determined above. For the fast diffusing component (*D* about 10^–10^ m^2^/s) of polymeric micelles, the analysis yielded particle sizes ranging from 1.7 nm (ZnPc-*t*-but_4_-loaded mPEG-*b*-PDLLA micelles, see Table 3) to 2.6 nm (empty mPEG-*b*-PCL and mPEG-*b*-PDLLA micelles, see Table S1) and, hence, may be attributed to unimers. The particle hydrodynamic diameters obtained for the slow diffusing population (*D* about 10^–11^ m^2^/s) ranged from about 24 nm (empty mPEG-*b*-PDLLA micelles, see Table S1) to about 39 nm (ZnPc-*t*-but_4_-loaded mPEG-*b*-PCL micelles, see Table 3) and were comparable to the mean polymeric micelle sizes reported by DLS (see Tables S2 and S3).^32^ The measured self-diffusion coefficients provide further evidence of the PM formation and their mean diameters. The diffusion coefficients and molecular diameters obtained for ZnPc-*t*-but_4_ in PMs were very similar for both block copolymers and equal to about 9.2 × 10^–10^ m^2^/s and 0.22 nm, respectively (see Table 2). The diffusion coefficients of ZnPc-*t*-but_4_ were around one order of magnitude higher (10^–10^ m^2^/s in comparison to 10^–11^ m^2^/s) than the *D* values of PMs, assuming higher diffusibility of Ps in comparison to nanocarriers.^26^ The aforementioned difference is strictly connected with the mean hydrodynamic diameters of free ZnPc-*t*-but_4_ single molecules (*ca*. 0.2 nm) and polymeric micelle (between 25 and 40 nm)—see Figure 1 and Tables 2, 3 and S1–S3.^24,32^ Therefore, values of diffusion coefficients for ZnPc-*t*-but_4_ in PMs indicate that phthalocyanine molecules experience free diffusion and do not undergo complexation by polymer chains in the core microenvironment. The complexation effects, mostly connected with hydrogen bonding, were observed, for example, for vanillin in the cyclodextrin solution, resulting in a concentration-sensitive change of diffusion coefficient values.^44^ Thus, the studied systems—ZnPc-*t*-but_4_ loaded PMs—are different due to the lack of hydrogen bonding in the core microenvironment. On the other hand, the micelle surface may constitute a barrier for ZnPc-*t*-but_4_ molecule diffusion outside the micelles into aqueous solution (no sign of phthalocyanine aggregation, which is very fast and widespread in the extra micellar environment).^12−14,26^ The diffusometry results of ZnPc-*t*-but_4_ were consistent with those of the UV–vis spectroscopy experiments, indicating the presence of the solubilized Ps in a monomeric (nonaggregated) form.

**Table 3:** Diffusion Coefficients (*D*_1_ and *D*_2_) and Calculated Hydrodynamic Diameters (Denoted 2*R*_h_) for the Two Populations in ZnPc-*t*-but_4_-Loaded mPEG-*b*-PCL and mPEG-*b*-PDLLA Micelles as Well as Hydrodynamic Diameters Obtained from DLS Experiments

	polymeric micelles		nonaggregated copolymer		
block copolymer	*D*_1_ × 10^–11^ m^2^/s	2*R*_h_ (nm)	*D*_2_ × 10^–10^ m^2^/s	2*R*_h_ (nm)	DH (nm) by DLS
mPEG-*b*-PCL	1.051 ± 0.037	38.1 ± 1.3	1.848 ± 0.103	2.2 ± 0.1	39.3 ± 0.3
mPEG-*b*-PDLLA	1.448 ± 0.195	27.7 ± 3.7	2.370 ± 0.231	1.7 ± 0.2	26.7 ± 1.4

### 1D NOE NMR Approach to Hydrophobic Probe Solubilization

In general, the solubilizate location in any type of nanocarrier is one of the most significant physicochemical properties and influences their stability, protection, loading efficiency, and release rate. Payloads that are physically entrapped in the nanocarrier interior are more stable and better protected against inactivation in contrast to those solubilized within the external parts of a nanocarrier. Generally, many phthalocyanines are hardly soluble in any solvents, due to their planar structure with limited accessibility of binding groups, but they tend to accumulate at the interfaces.^12^ The aforementioned phenomena may result in a significant loss of photoactivity when phthalocyanine molecules are positioned on the external surface of a nanocarrier or the corona-core interface, being readily accessible to an aqueous environment. The most possible mechanism involves spontaneous aggregation, followed by the formation of nonphotoactive forms of the Ps. On the other hand, phthalocyanine molecules may accumulate on the internal borders between different microphases inside micellar cores, that is, rigid crystalline, rigid amorphous, or flexible amorphous, resulting in low susceptibility to the aggregate and profound photoactivity due to the very slow motion and diffusion in polymeric domains as well as separation from the aqueous environment.

As the NOE describes a phenomenon of a certain spin population relaxation, influencing, via through-space interactions, the energy levels of other spins (i.e., their signal intensities in the spectrum), it is particularly sensitive for analysis of physically mixed compounds.^18,34^ The increase or decrease of the signal intensity is dependent on the tumbling properties and is visible as the respective positive (generally for small molecules) or negative (generally for large molecules) peak in the NOE spectrum.^24^ As it was aforementioned, the NMR spectroscopy with the NOE can be used to infer the location of a solute in a polymeric micelle or matrix by the selective saturation of the payload and/or block copolymer or surfactant protons and intermolecular interaction analysis. According to ^1^H NMR and *T*_1_ relaxation times (the supposed presence of the solubilizate in the core of PMs) for selective saturation, intensive signals of the hydrophobic parts were chosen, that is, methylene protons f at 4.037 ppm (for PCL) and methyl protons c at 1.598 ppm (for PDLLA). Significant differences in chemical shifts as well as *T*_1_ relaxation times (see Table 2) were observed for the aforementioned signals between ZnPc-*t*-but_4_-loaded and empty micelles. The NOE NMR experiment is fully consistent with NMR relaxometry experiments and directly shows the spatial proximity between the phthalocyanine probe and appropriate core-forming polymers (PCL or PDLLA). Moreover, the aforementioned experiments enabled to confirm the solubilization locus of ZnPc-*t*-but_4_ in the PM core, hypothesized from its high hydrophobicity and UV–vis spectra.

**Figure 3:**
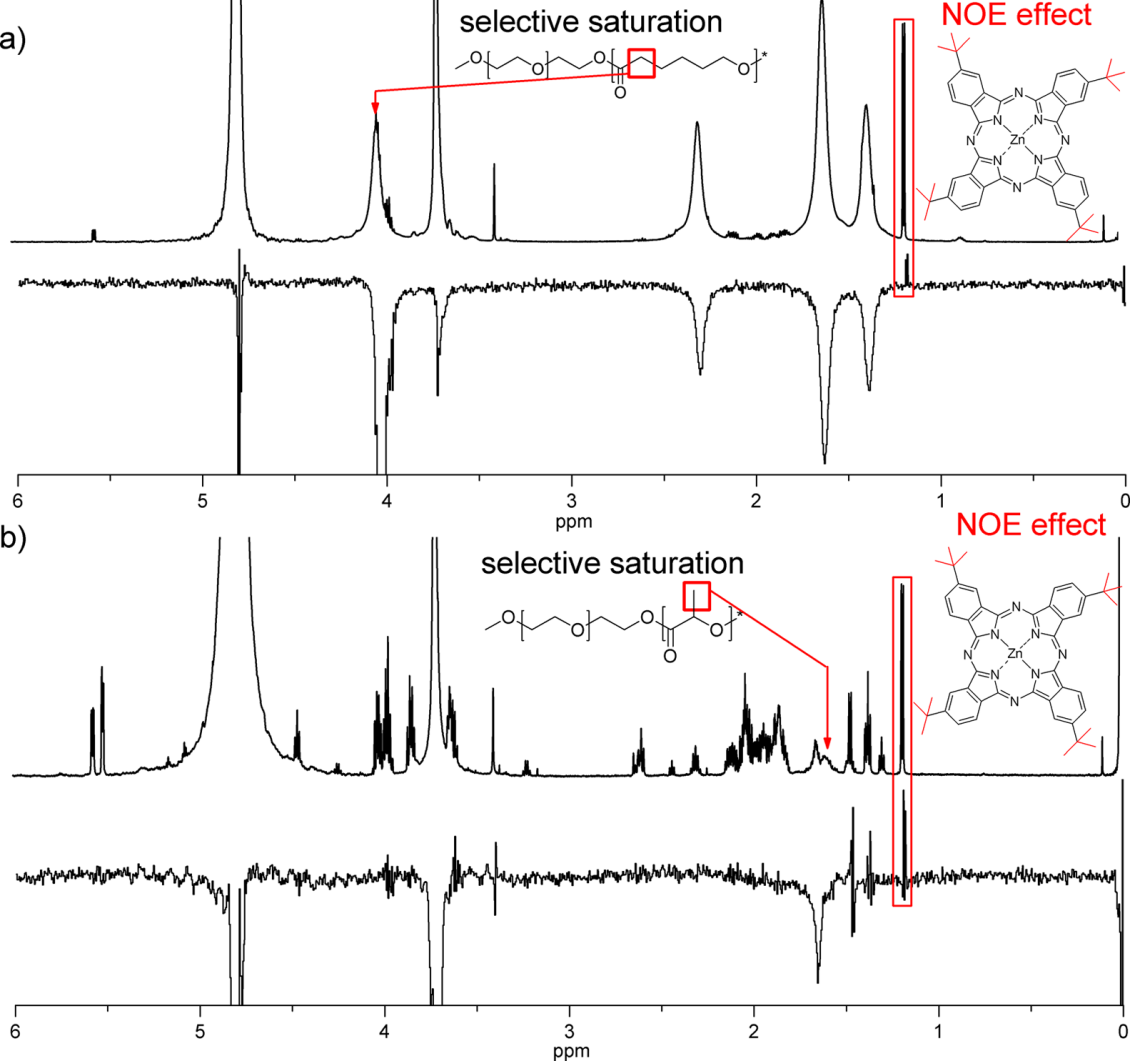
1D NOE spectra (upper panel, 0–6 ppm region) of mPEG-*b*-PCL (a) and mPEG-*b*-PDLLA (b) micelles loaded with ZnPc-*t*-but_4_ obtained upon selective saturation of the marked signal (attributed to the selected protons of the hydrophobic chain) with the reference ^1^H NMR spectra (lower panel, 0–6 ppm region).

As shown in Figure 3, the presence of the marked signals at 1.175 and 1.186 ppm obtained by the selective saturation of methylene (in case of ZnPc-*t*-but_4_-loaded mPEG-*b*-PCL micelles, chemical shift 4.037 ppm) and methyl (in case of ZnPc-*t*-but_4_-loaded mPEG-*b*-PDLLA micelles, chemical shift 1.598 ppm) protons, respectively, undeniably confirmed the interaction between the solute and hydrophobic fragment of the block copolymer. The aforementioned signals are positive, due to the relatively low-molecular weight of ZnPc-*t*-but_4_ in comparison to the polymers. The NOE investigations as well as ^1^H NMR and *T*_1_ relaxation time analysis showed that phthalocyanine molecules are located within the core of the PMs. The selectively saturated signals in mPEG-*b*-PCL micelles (methylene protons at 4.037 ppm) were found to have spatial proximity with all other methylene groups in the PCL chain (chemical shifts: 2.296, 1.622, and 1.382 ppm)—strong negative signal (large molecule—interactions within the block copolymer)—due to the formation of the core structure with flexible polymeric subdomains. The aforementioned saturated methylene group is also situated in spatial proximity with PEG protons (chemical shift 3.718 ppm), although the signal (also negative due to the polymeric character of the molecule) is significantly weaker in comparison to other methylene moieties in the PCL chain, counterintuitively to the large number of protons, constituting the PEG fragment. Moreover, no NOE signal was observed for the methyl group at the end of the PEG chain (chemical shift 3.398 ppm) upon the saturation of methylene protons at 4.037 ppm. The obtained results suggest that the corona shell of PM–PEG chains extends into the aqueous environment and is separated from the core-forming PCL fragments; the observed weak interactions between PCL and PEG protons may be easily explained by the formation of a thin interphase between the corona and the core. The selective saturation of methyl protons in the PDLLA chain (chemical shift 1.598 ppm) gave a distinct NOE response for two signals: strong positive for PEG protons (chemical shift 3.718 ppm) as well as the aforementioned negative peak for *tert*-butyl protons in ZnPc-*t*-but_4_ (chemical shift 1.175–1.186 ppm). A strong positive response for the PEG signal may be explained via inter- and intramolecular interactions at the corona-core interface as well as the amorphous structure of the micelle core. On the other hand, in contrast to PCL, there are no visible NOE signals for methine groups in the PDLLA chain (chemical shift 5.294 ppm) upon the saturation of methyl protons (chemical shift 1.598 ppm). This fact is possibly connected to a large difference (and high value) of spin–lattice relaxation times between methyl (2.906 s) and methine (4.120 s) protons, resulting in insufficient probability to generate a rapidly oscillating field, required for the NOE effect.^26^

### Impact of the Core-Type Microenvironment on the Photochemical Performance of Tetra-*tert*-butyl zinc(II) Phthalocyanine

It has to be emphasized that the main role of the photoactive compound nanocarrier is the significant reduction of any unwanted photoprocesses like photobleaching, resulting in an irreversible loss of activity, as well as providing the optimal microenvironment for the desired photoreaction. For tetra *tert*-butyl zinc(II) phthalocyanine intended to act as a Ps, the main process comprises the generation of singlet oxygen via the irradiation of its photoactive form, followed by ^1^O_2_ diffusion from the nanocarrier to the actual place of action (e.g., the diseased tissue or, for use as a probe, solution of the appropriate singlet oxygen scavenger). Generally, the second possibility—the release of the Ps molecules from the nanocarrier, followed by the irradiation of the photoactive form—is impossible for the highly hydrophobic ZnPc-*t*-but_4_ due to its very low aqueous solubility and tendency to aggregate in a polar environment.^12,13^ Very low aqueous solubility of ZnPc-*t*-but_4_ makes it difficult to study any release without the addition of hydrotropic compounds like benzoic acid or Tween-type surfactants. The release studies for phthalocyanines most often involves the use of appropriate surfactants or hydrotropic molecules in order to prevent aggregation, loss of photoactivity, and precipitation of Ps molecules.^12−15^ Moreover, PMs loaded with phthalocyanine-type dyes may act as specific photocatalysts in aqueous systems.

**Figure 4:**
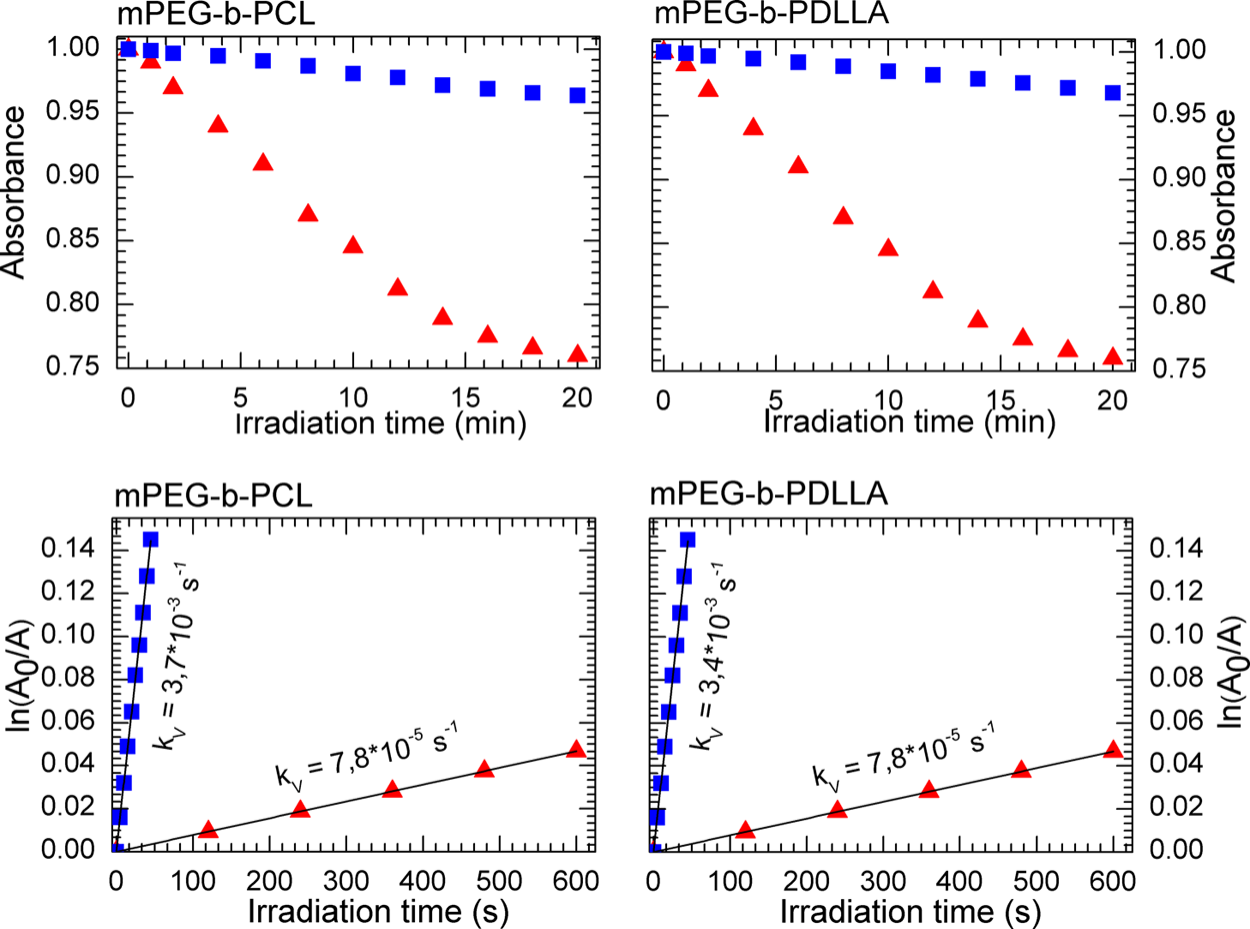
Photobleaching (upper panel) and singlet oxygen generation (lower panel) by ZnPc-*t*-but_4_ in aqueous solution (red triangles) as well as in mPEG-*b*-PCL and mPEG-*b*-PDLLA micelles (blue squares).

The photochemical properties of ZnPc-*t*-but_4_ (i.e., photobleaching and ^1^O_2_ generation rates) were measured in both its native state (in 1% PEG water solution) and loaded in mPEG-*b*-PCL and mPEG-*b*-PDLLA micelles. The photobleaching process was presented by plotting the change in absorbance (in arbitrary units) versus irradiation time (Figure 4, upper panel). The achieved data clearly proved that the encapsulated ZnPc-*t*-but_4_ (within hydrophobic nanodomains of the PMs) showed better photostability during irradiation in regard to the native form of the Ps, which is in very good agreement with the observations of ZnPc-loaded mPEG-*b*-PLLA micelles presented in our previous studies.^4,34^ Moreover, no significant difference between the photobleaching of ZnPc-*t*-but_4_ in semicrystalline (mPEG-*b*-PCL) and amorphous (mPEG-*b*-PDLLA) microenvironments was observed. The aforementioned effect is connected with the mechanism of the photobleaching process, involving multistep changes in the chromophore moiety initiated by self-oxidation by the generated singlet oxygen. In both systems, the probe molecules are accumulated within the polymeric matrix, so some significant chemical changes (e.g., disruption of the planar structure) may not be possible due to partially restricted mobility at subdomain borders. Moreover, the aforementioned photostability of ZnPc-*t*-but_4_ in the hydrophobic nanodomains of PCL and PDLLA is consistent with an optimal, hydrophobic environment as well as significantly reduced susceptibility to undergo aggregation, resulting in the loss of phthalocyanine activity.

The first-order rate constants of ^1^O_2_ generation were determined in the present contribution by means of photobleaching of 9,10-anthracenediyl-bis(methylene)dimalonic acid sodium salt, that is, ABMDMA, under the irradiation of free and encapsulated ZnPc-*t*-but_4_ in the presence of oxygen (see Figure 4, lower panel). This indirect method was used because the ^1^O_2_ phosphorescence, which is another indicator of the presence of the singlet oxygen, is difficult to observe in water solutions.^2^ The obtained results demonstrate that the chemical probe ABMDMA was much more effective, that is, two orders of magnitude better; *k*_v_ = 3.7 × 10^–3^ s^–1^ (in mPEG-*b*-PCL) or *k*_v_ = 3.4 × 10^–3^ s^–1^ (in mPEG-*b*-PDLLA) in comparison to *k*_v_ = 7.8 × 10^–5^ s^–1^, oxidized by the encapsulated ZnPc-*t*-but_4_ than by its free form. A very similar phenomenon was observed for ZnPc encapsulated in mPEG-*b*-PLLA micelles^34^ prepared in a slightly different manner (interfacial deposition—co-solvent evaporation method instead of the thin-film approach). The calculated values of *k*_v_ constants for ZnPc-*t*-but_4_-loaded in mPEG-*b*-PCL (3.7 × 10^–3^ s^–1^) and mPEG-*b*-PDLLA (3.4 × 10^–3^ s^–1^) micelles indicate that the core state (semicrystalline or amorphous) has a low impact on the process of singlet oxygen generation. Thus, the obtained photobleaching and ^1^O_2_ generation data remain satisfactorily in line with our other studies,^33,34^ and the excellent photochemical properties of ZnPc-loaded mPEG-*b*-PLLA micelles may be ascribed to the active compound located within the hydrophobic micellar core. The aforementioned effect is most probably directly linked with the relatively long lifetime (*ca.* 10^–11^ to 10^–3^s), enabling their easy diffusion even at a distance as long as a few hundreds of nanometers. Moreover, the structure of the core microenvironments, comprising subdomains of different flexibility and their interfaces, may act as channels for the singlet oxygen diffusion outside the PMs. The photobleaching studies are congruent with the ^1^O_2_ generation results, that is, the encapsulation of ZnPc postpones photobleaching and enhances ROS production. Both of these features are strictly related to the appropriate properties of the PM core microenvironment. Our findings in the fields of colloidally and photochemically stable ZnPc-*t*-but_4_-loaded mPEG-*b*-PCL and ZnPc-*t*-but_4_–mPEG-*b*-PDLLA micelles constitute new opportunities especially for PDT, nanotheranostic application, as well as combination therapeutics because the reduced photobleaching rates and profound singlet oxygen generation abilities, together with optimal dimensions of corona-core nanocarriers, are the most important features of Pss’ formulations for the aforementioned anticancer modality.^45,46^

## Conclusions

The amphiphilic block copolymers self-assembly in aqueous systems into corona-core structures, that is,, PMs, characterized not only by two main microenvironments: hydrophilic and hydrophobic, but also numerous subdomains of different crystallinities and rigidities. The aforementioned structures may be studied by probing with appropriate compounds, miscible with the particular microphases, and selectively changing their spectroscopic properties, for example, UV–vis spectra, fluorescence emission/absorption bands, NMR shifts, *T*_1_ and/or *T*_2_ relaxation times, and diffusibility. Subdomains in PMs’ systems play a crucial role in their usefulness toward their application as nanocarriers, especially for biologically active compounds, optical probes, diagnostic agents, and Pss, influencing their controlled release profiles, photostability, as well as the ability to generate ROS. The applied multifunctional photoactive probe—tetra *tert*-butyl zinc(II) phthalocyanine (ZnPc-*t*-but_4_)—providing unique optical and magnetic resonance properties is one of the most suitable choices for probing subtle polymeric microenvironments.

The ZnPc-*t*-but_4_-loaded mPEG-*b*-PCL and mPEG-*b*-PDLLA micelles exhibited good physical stability, high active compound loading content, and a size of less than *ca*. 50 nm with low polydispersity indices; the parameters that meet the necessary requirements for PM drug delivery systems. ^1^H NMR and 1D NOE analyses demonstrated that ZnPc-*t*-but_4_ molecules are in spatial proximity with the polyester hydrophobic chains of both block copolymers. In addition, they indicated that the probe was entrapped within the polymeric matrix, forming the micellar core. Those findings are fully consistent with the solubility and miscibility parameter approach, indicating the formation of the corona (PEG)—core (PCL or PDLLA) micelle systems with phthalocyanine molecules compatible with the internal, hydrophobic domain. The values of the hydrodynamic diameter of PMs obtained by ^1^H NMR diffusometry were consistent with the mean PMs’ sizes reported by DLS. UV–vis spectroscopy and ^1^H NMR with diffusometry/relaxometry analysis revealed differences between the microenvironments of mPEG-*b*-PCL and mPEG-*b*-PDLLA cores as well as confirmed the photoactive probe solubilization in a monomeric form.

The NMR probing as well as UV–vis measurements suggest that, in order to prevent the loss of phthalocyanine photoactivity, the structure of PMs’ cores (PCL or PDLLA) should contain at least two subdomains of different flexibilities. Such a microenvironment is an optimal space for planar, hydrophobic molecules, which exploit their high susceptibility to accumulate at hydrophobic interfaces and reveal some restricted probability to move and aggregate in the polymer matrix. On the other hand, planar compounds entrapped within such microenvironments, for example, ZnPc-*t*-but_4_, are characterized by the noticeably reduced probability of unwanted loss of photoactivity, whereas the diffusion of singlet oxygen (^1^O_2_) from the polymeric matrix to external aqueous solution is not restricted. Thus, our findings might be especially interesting for the potential use of hydrophobic phthalocyanine—type pharmaceuticals (e.g., Ps for PDT, diagnostic agents in nanotheranostics, components for combination drug delivery) or photocatalysts in aqueous systems. The present contribution may open a new possibility of selecting more suitable nanoscale polymeric matrices as host materials for functional polymeric dispersions, indicating the most important features for consideration in their future research.

## Supporting Information Available

Data of PM size and their distribution by DLS and DOSY NMR; PM colloidal stability by DLS; PM raw DLS data; solubilization characterization of ZnPc-*t*-but_4_ in mPEG-*b*-PCL and mPEG-*b*-PDLLA micelles; solubility and miscibility parameters for ZnPc-*t*-but_4_ in mPEG-*b*-PCL and mPEG-*b*-PDLLA micelles; and determination of diffusion coefficients and hydrodynamic diameters for PMs (PDF)
