# Supplementary material for: Studying the “Rigid–Flexible” Properties of Polymeric Micelle Core-Forming Segments with a Hydrophobic Phthalocyanine Probe Using NMR and UV Spectroscopy
Source: Langmuir. 2021 Apr 2;37(14):4316–30. doi: 10.1021/acs.langmuir.1c00328 (PMC8154882; doi:10.1021/acs.langmuir.1c00328)
Supplement: Supplementary file 1 — la1c00328_si_001.pdf [file la1c00328_si_001.pdf]

Supporting Information

Studying the „Rigid-Flexible" Properties of Polymeric Micelles Core-Forming Segments by a Hydrophobic Phthalocyanine Probe using NMR and UV Spectroscopies

Łukasz Lamch*, Roman Gancarz, Marta Tsirigotis-Maniecka, Izabela Moszyńska, Justyna Ciejka, Kazimiera A. Wilk*

Department of Engineering and Technology of Chemical Processes, Faculty of Chemistry, Wrocław University of Science and Technology, Wybrzeże Wyspiańskiego 27, 50-370 Wrocław, Poland

* To whom correspondence should be addressed

phone: +48 71 3203447, fax: +48 71 3203678

email addresses: lukasz.lamch@pwr.edu.pl, kazimiera.wilk@pwr.edu.pl

Table of contents Page

1. Polymeric micelles size and its distribution by DLS and DOSY NMR S2

2. Polymeric micelles colloidal stability by DLS S2

3. Polymeric micelles – raw DLS data S3

4. Solubilization of ZnPc-*t*-but$_4$ in mPEG-*b*-PCL and mPEG-*b*-PDLLA micelles S4

5. Solubility and miscibility parameters for ZnPc-*t*-but$_4$ in mPEG-*b*-PCL and mPEG-*b*-PDLLA micelles S6

6. Determination of diffusion coefficients and hydrodynamic diameters for polymeric micelles S7

1. Polymeric micelles size and its distribution by DLS and DOSY NMR

Table S1. Diffusion coefficients (D_1 and D_2) and calculated hydrodynamic diameters (denoted as 2R$_h$) for the two populations of empty mPEG-*b*-PCL and mPEG-*b*-PDLLA micelles as well as hydrodynamic diameters obtained from DLS experiments.

Block copolymer	polymeric micelles		non aggregated copolymer		D_H (nm) by DLS
	$D_1 \times 10^{-11}$ m^2/s	2R$_h$ (nm)	$D_2 \times 10^{-10}$ m^2/s	2R$_h$ (nm)	
mPEG-*b*-PCL	1.104±0.011	36.2±0.4	1.537±0.026	2.6±0.1	38.0±1.1
mPEG-*b*-PDLLA	1.671±0.079	23.9±1.1	1.551±0.023	2.6±0.1	25.8±0.5

2. Polymeric micelles colloidal stability by DLS

Table S2. Colloidal stability of the studied polymeric micelles without payload.

System	Block copolymer	[copolymer] (mg/cm^3)	D_H (nm)	PdI
		t = 0		
1a	mPEG-*b*-PCL	6.7	38.0±1.1	0.09±0.02
2a	mPEG-*b*-PDLLA	6.7	25.8±0.5	0.27±0.01
		t = 30 days		
1a	mPEG-*b*-PCL	6.7	39.0±0.5	0.12±0.01

2a	mPEG-*b*-PDLLA	6.7	25.3±1.6	0.26±0.01
		t = 60 days		
1a	mPEG-*b*-PCL	6.7	39.6±0.7	0.15±0.01
2a	mPEG-*b*-PDLLA	6.7	27.7±2.1	0.25±0.01

3. Polymeric micelles – raw DLS data

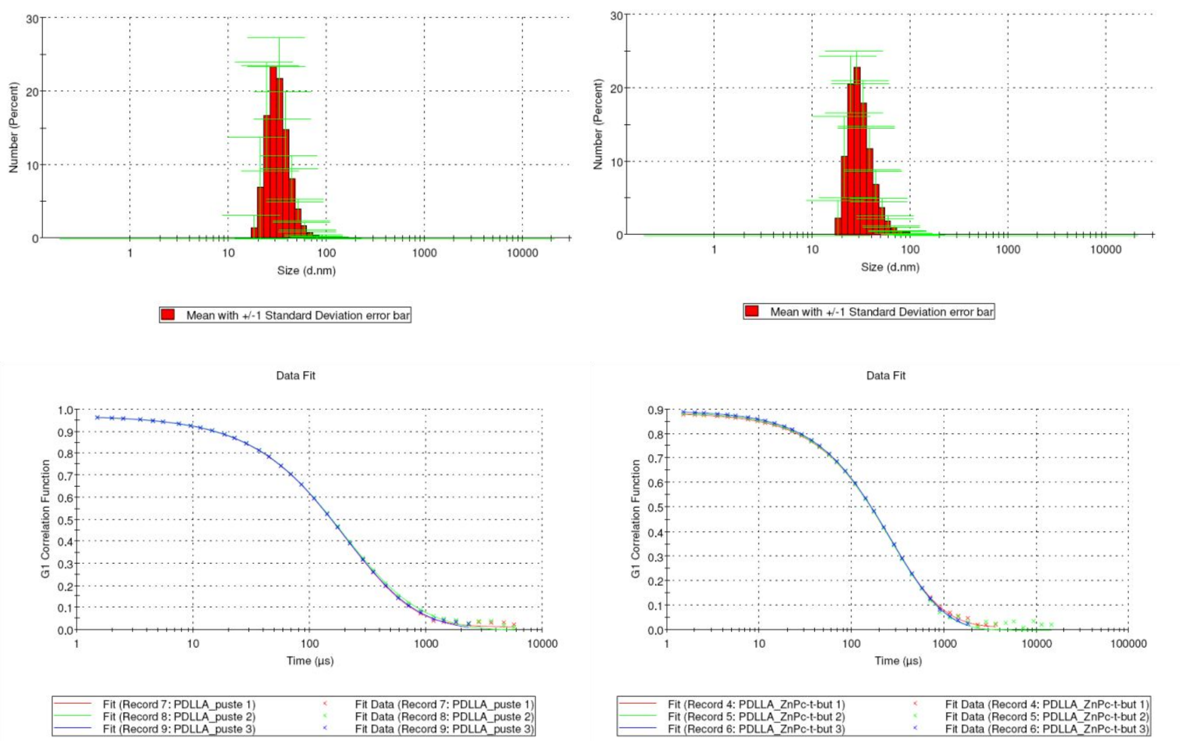

Figure S1. Autocorrelation functions and size statistic reports by number (raw data) for empty (left) and ZnPc-*t*-but$_4$-loaded (right) mPEG-*b*-PDLLA micelles.

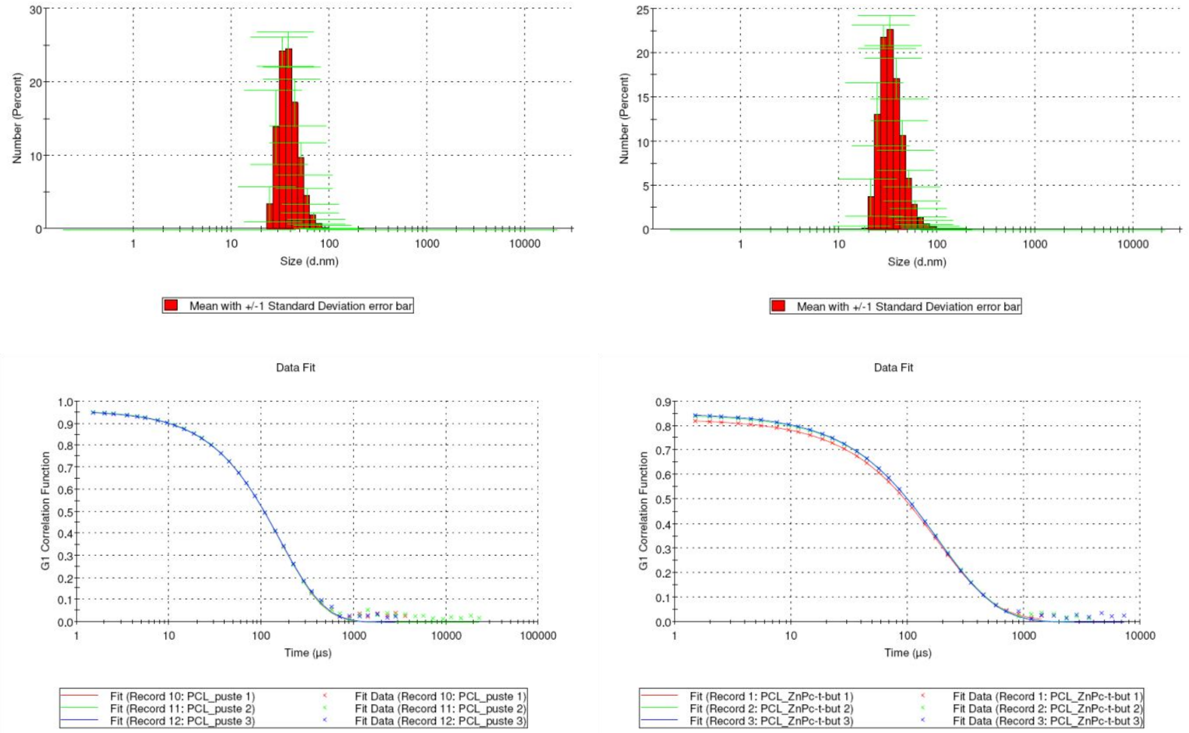

Figure S2. Autocorrelation functions and size statistic reports by number (raw data) for empty (left) and ZnPc-*t*-but$_4$-loaded (right) mPEG-*b*-PCL micelles.

4. Solubilization of ZnPc-t-but$_4$ in mPEG-b-PCL and mPEG-b-PDLLA micelles

The ZnPc-*t*-but$_4$ concentration in polymeric micelles ([ZnPc-*t*-but$_4$]$_{micelle}$) was calculated from Lambert-Beer equation. while the percentage loading efficiency was determined by the following formula:

$$\%\ \text{loading efficiency} = \frac{[\text{ZnPc - t - but}_4]_{\text{micelle}}}{[\text{ZnPc - t - but}_4]_{\text{added}}}\ 100\% \qquad (1)$$

The highest [ZnPc-*t*-but$_4$]$_{micelle}$ at which 100% loading efficiency was achieved (max[ZnPc-*t*-but$_4$]$_{micelle100\%}$) was used to calculate the maximum concentration of phthalocyanine contained in micelles (max[ZnPc-*t*-but$_4$]$_{micelle}$) by

$$\max[\text{ZnPc-}t\text{-but}_4]_{micelle} = \max[\text{ZnPc-}t\text{-but}_4]_{micelle\ 100\%} - [\text{ZnPc-}t\text{-but}_4]_{aqueous} \qquad (2)$$

For all phthalocyanines $[\text{ZnPc-}t\text{-but}_4]_{aqueous}$ << $\max[\text{ZnPc-}t\text{-but}_4]_{micelle\ 100\%}$ so $\max[\text{ZnPc-}t\text{-but}_4]_{micelle} \approx \max[\text{ZnPc-}t\text{-but}_4]_{micelle\ 100\%}$ $[\text{ZnPc-}t\text{-but}_4]_{aqueous}$ was calculated by ChemAxon software for neutral pH and was found to be equal to 2.45×10^{-14} mg/mL.

Percentage Ps/polymer ratio (described by wt%) was calculated by the following formula:

$$\% \text{ Ps/polymer ratio} = \frac{\max[\text{ZnPc - t - but}_4]_{\text{micelle}}}{[\text{polymer}]_{\text{micelle}}} 100\% \qquad (3)$$

The polymer concentration in micelles ($[\text{polymer}]_{micelle}$) was calculated by:

$$[\text{polymer}]_{\text{micelle}} = [\text{polymer}]_{\text{added}} - \text{CMC} \qquad (4)$$

The polymer concentrations $[\text{polymer}]_{added}$ (6.7 mg/mL for mPEG-*b*-PCL and mPEG-*b*-PDLLA) were about three orders of magnitude higher than CMC value of mPEG-*b*-PCL and mPEG-*b*-PDLLA copolymers (4.5×10^{-3} mg/mL and 1.0×10^{-3} mg/mL. respectively) so $[\text{polymer}]_{micelle} \approx [\text{polymer}]_{added}$.

The partition coefficients of each phthalocyanines in polymeric micelles at their $\max[\text{ZnPc-}t\text{-but}_4]_{micelle}$ were determined using the equation:

$$\frac{[\text{ZnPc - t - but}_4]_{\text{micelle}}}{[\text{ZnPc - t - but}_4]_{\text{aqueous}}} = P\,X_{polymer}\frac{C}{\rho} \qquad (5)$$

where *P* is the partition coefficient of the drug. *C* the concentration of copolymer (6.7×10^{-3} g/mL). $X_{polymer}$ – the mole fraction of hydrophobic part in block copolymer (0.438 for mPEG-*b*-PCL and 0.554 for mPEG-*b*-PDLLA) and ρ the bulk density of PCL and PDLLA (1.145 and 1.250 g/mL. respectively).

Table S3. **Composition, payload/block copolymer ratio, loading efficiency, polymeric micelles-water partition coefficient (logP), hydrodynamic diameter (D_H) and its distribution (PdI) of the studied polymeric micelles.**

System	Block copolymer	[copolymer]	$[\text{ZnPc-}t\text{-but}_4]_{micelle}$	ZnPc-*t*-but$_4$/	Loading	logP	D_H (nm)	PdI

		(mg/mL)	(μM)	polymer ratio (%)	efficiency (%)			
				t = 0				
1b	mPEG-*b*-PCL	6.7	102.8±2.7	15.3±1.2	100±9	15.3	39.3±0.3	0.13±0.01
2b	mPEG-*b*-PDLLA	6.7	67.4±1.8	10.0±0.7	92±9	15.2	26.7±1.4	0.27±0.02
				t = 30 days				
1b	mPEG-*b*-PCL	6.7	103.4±2.1	15.3±1.2	99±10	15.3	38.4±1.4	0.10±0.01
2b	mPEG-*b*-PDLLA	6.7	67.1±1.6	10.0±0.7	100±11	15.2	25.0±0.7	0.28±0.01
				t = 60 days				
1b	mPEG-*b*-PCL	6.7	102.1±2.3	15.3±1.2	100±9	15.3	37.6±1.2	0.11±0.01
2b	mPEG-*b*-PDLLA	6.7	66.8±2.0	10.0±0.7	100±9	15.2	25.9±0.9	0.29±0.02

5. Solubility and miscibility parameters for ZnPc-t-but$_4$ in mPEG-b-PCL and mPEG-b-PDLLA micelles

Solubility parameters (δ_x) for the studied block copolymers (mPEG-*b*-PCL and mPEG-*b*-PDLLA) and probe (ZnPc-*t*-but$_4$) were calculated Hoy's group increment method. utilizing values of cohesive energy (E_{coh}) group contributions and molar volumes (V) provided by Fedors:

$$\delta_x = \sqrt{\frac{E_{coh}}{V}} \qquad (6)$$

The calculated values of solubility parameters for particular blocks of copolymers (PEG. PCL and PDLLA) and payload (ZnPc-*t*-but$_4$). denoted as δ_p and δ_s, respectively, were used for calculation of miscibility parameter (Flory-Huggins interaction parameter – χ):

$$\chi = (\delta_p - \delta_s)^2 \frac{V}{RT} \qquad (7)$$

Where V is molar volume of the payload. R – the universal gas constant and T – temperature in Kelvins.

Table S4. Solubility parameters (δ_p) of particular polymers' fragments and miscibility (χ) parameters between polymers' fragments and water (δ_a equal to 47.9 MPa$^{0.5}$).

Polymer fragment	E_{coh} [J/mol]	V [cm^3/mol]	δ_p [MPa$^{0.5}$]	χ
PEG	13230	36.0	19.2	12.0
PCL	42700	98.5	20.9	29.2
PDLLA	26140	50.5	22.9	12.9

Table S5. Solubility parameters (δ_p) of particular polymers' fragments and miscibility (χ) parameters between polymers' fragments and ZnPc-*t*-but$_4$ (δ_s equal to 22.7 MPa$^{0.5}$).

Polymer fragment	E_{coh} [J/mol]	V [cm^3/mol]	δ_p [MPa$^{0.5}$]	χ
PEG	13230	36.0	19.2	2.45
PCL	42700	98.5	20.9	0.70
PDLLA	26140	50.5	22.9	0.01

6. Determination of diffusion coefficients and hydrodynamic diameters for polymeric micelles

Monoexponentional function (one diffusion coefficient):

$$A = A_1 \exp\left(-D_1\left(\Delta - \frac{\delta}{3}\right)(\delta\,\gamma\,G_i)^2\right)$$

Biexponentional function (two diffusion coefficients):

$$A = A_1 \exp\left(-D_1\left(\Delta - \frac{\delta}{3}\right)(\delta\,\gamma\,G_i)^2\right) + A_2 \exp\left(-D_2\left(\Delta - \frac{\delta}{3}\right)(\delta\,\gamma\,G_i)^2\right)$$

Triexponentional function (three diffusion coefficients):

$$A = A_1 \exp\left(-D_1\left(\Delta - \frac{\delta}{3}\right)(\delta\,\gamma\,G_i)^2\right) + A_2 \exp\left(-D_2\left(\Delta - \frac{\delta}{3}\right)(\delta\,\gamma\,G_i)^2\right) +$$

$$+ A_3 \exp\left(-D_3\left(\Delta - \frac{\delta}{3}\right)(\delta\,\gamma\,G_i)^2\right)$$

Table S6. Determination of diffusion coefficients (*D*) and hydrodynamic diameters (D_H) by fitting appropriate models to data: G^1 (intensity versus linear gradient) or G^2 (intensity versus square gradient). Bold – the best fit.

Block copolymer	Model (G^1 or G^2)	empty			+ZnPc-*t*-but$_4$		
		D [m^2/s]	D_H [nm]	R^2	*D* [m^2/s]	D_H [nm]	R^2
mPEG-*b*-PCL	G^1 (1 coefficient)	1.950×10^{-11}	20.5	0.91949	1.974×10^{-11}	20.3	0.88651
	G^1 (2 coefficients)	**1.537×10^{-10}**	**2.6**	**0.99994**	**1.849×10^{-10}**	**2.2**	**0.99907**
		**1.104×10^{-11}**	**36.3**		**1.051×10^{-11}**	**38.1**	
	G^1 (3 coefficients)	1.666×10^{-10}	2.4	0.99996	1.818×10^{-9}	0.2	0.99993
		3.739×10^{-11}	10.7		1.539×10^{-10}	2.6	
		9.920×10^{-12}	40.4		1.003×10^{-11}	39.9	
	G^2 (1 coefficient)	1.947×10^{-11}	20.6	0.91949	1.979×10^{-11}	20.2	0.88651
	G^2 (2 coefficients)	**1.537×10^{-10}**	**2.6**	**0.99994**	**1.848×10^{-10}**	**2.2**	**0.99907**
		**1.104×10^{-11}**	**36.3**		**1.051×10^{-11}**	**38.1**	
	G^2 (3 coefficients)	1.666×10^{-10}	2.4	0.99996	1.817×10^{-9}	0.2	0.99993
		3.739×10^{-11}	10.7		1.539×10^{-10}	2.6	
		9.918×10^{-12}	40.4		1.003×10^{-11}	39.9	
mPEG-*b*-PDLLA	G^1 (1 coefficient)	9.082×10^{-11}	4.4	0.96869	5.738×10^{-11}	7.0	0.86565
	G^1 (2 coefficients)	**1.551×10^{-10}**	**2.6**	**0.99990**	**2.382×10^{-10}**	**1.7**	**0.99477**
		**1.666×10^{-11}**	**24.0**		**1.444×10^{-11}**	**27.7**	
	G^1 (3 coefficients)	1.560×10^{-10}	2.6	0.99988	2.053×10^{-09}	0.2	0.99988
		-6.812×10^{-11}	-5.9		1.644×10^{-10}	2.4	
		1.728×10^{-11}	23.2		1.152×10^{-11}	34.7	
	G^2 (1 coefficient)	1.233×10^{-10}	3.3	0.99644	5.737×10^{-11}	7.0	0.86565
	G^2 (2 coefficients)	**1.551×10^{-10}**	**2.6**	**0.99990**	**2.380×10^{-10}**	**1.7**	**0.99477**
		**1.671×10^{-11}**	**24.0**		**1.448×10^{-11}**	**27.7**	
	G^2 (3 coefficients)	1.551×10^{-10}	2.6	0.99988	2.052×10^{-9}	0.2	0.99988
		1.551×10^{-10}	2.6		1.644×10^{-10}	2.4	
		1.671×10^{-11}	24.0		1.152×10^{-11}	34.8	
